# Supplementary material for: Individual and neighborhood based socioeconomic factors relevant for contact behaviour and epidemic control
Source: Commun Med (Lond). 2025 Dec 11;6:26. doi: 10.1038/s43856-025-01282-y (PMC12808736; doi:10.1038/s43856-025-01282-y)
Supplement: Supplementary file 2 — Supplementary Material [file 43856_2025_1282_MOESM2_ESM.pdf]

Supplementary Information

Individual and neighbourhood based socioeconomic factors relevant for contact behaviour and epidemic control

Laura Di Domenico[1,+,*], Martina L. Reichmuth[1], Christian L. Althaus[1,2]

[1] *Institute of Social and Preventive Medicine, University of Bern, Bern, Switzerland*
[2] *Multidisciplinary Center for Infectious Diseases, University of Bern, Bern, Switzerland*

[+] Currently at Data Science Institute, Hasselt University, Hasselt, Belgium
* corresponding author: laura.didomenico@uhasselt.be

Table of contents

1. Supplementary Methods: data sources.......2
  1.1 Survey participants and survey waves.......2
  1.2 Weighted SEP index.......2
  1.3 Population size by age group, SEP level and education level.......4
2. Supplementary Results: contact determinants.......5
  2.1 Contact activity by survey wave.......5
  2.2 Regression model: sensitivity analyses.......6
3. Supplementary Methods: construction of contact matrices.......10
  3.1 Reciprocity correction.......10
  3.2 Matrix expansion.......10
    3.2.1 Inferring matrix elements in the diagonal blocks.......12
    3.2.2 Inferring matrix elements in the off-diagonal blocks.......15
  3.3 Assortativity index.......18
  3.4 Variation in dominant eigenvalue.......19
4. Supplementary Results: epidemic control.......22
  4.1 Type-reproduction number.......22
  4.2 Epidemic scenario with homogenous susceptibility.......22
5. Supplementary Results: validation.......24
6. Supplementary Results: alternative definition of education level for children.......27
Supplementary References.......31

1. Supplementary Methods: data sources

1.1 Survey participants and survey waves

The social contact data from Switzerland are presented in detail in another study[1]. To increase the sample size, we aggregated the adult participants from surveys categorized as A1, B1, and F1 (and for children, C1, D1 and E1), even though they refer to different periods, i.e., January - February 2021, June - July 2021 and December 2021 - January 2022, respectively. Survey periods are depicted in **Fig. S1**, together with the stringency index and variations in mobility to provide context in terms of non-pharmaceutical interventions. The period of January - February 2021 was characterized by a higher stringency index and a stronger variation in mobility, meaning that public health measures were stricter compared to the other two survey periods.

All participants in the survey reported the following information: age, gender, region (urban or rural), country of birth, education level, household income, employment status, household size, COVID-19 vaccination status, municipality of residence. We divided the participants into four age groups, i.e., 0-14, 15-24, 25-64, and 65+ year olds, to match available population data. We refer to these age groups as children, young adults, adults, and seniors, respectively. Age of children in the survey was reported in age-brackets. For simplicity, we assigned participants reported as 12-15 year olds to the age group of 0-14 year olds. For gender, we considered female, male, and other (the latter including answers such as ‘in another way’ or ‘prefer not to answer’). Country of birth was categorized as Switzerland, European Union, outside European Union, or unknown. We aggregated the education level into two categories: high education level, and middle-low education level. The former group corresponds to the tertiary level in the Swiss Education System[2], which includes advanced education or a university degree (Bachelor, Master or PhD); the latter group includes individuals with upper-secondary education or without any post-compulsory education. All children were classified as middle-low education. Monthly household income was grouped in four categories: 0-5,000 CHF, 5,001-10,000 CHF, 10,000+ CHF, and ‘prefer not to answer’. Employment status was classified in two groups, i.e., employed or unemployed, with the latter including the following answers: 'retired', 'student', 'homemaker', 'unemployed', 'other unemployed situation'. Household size was considered in three groups, as single units, couples or larger households with three or more members.

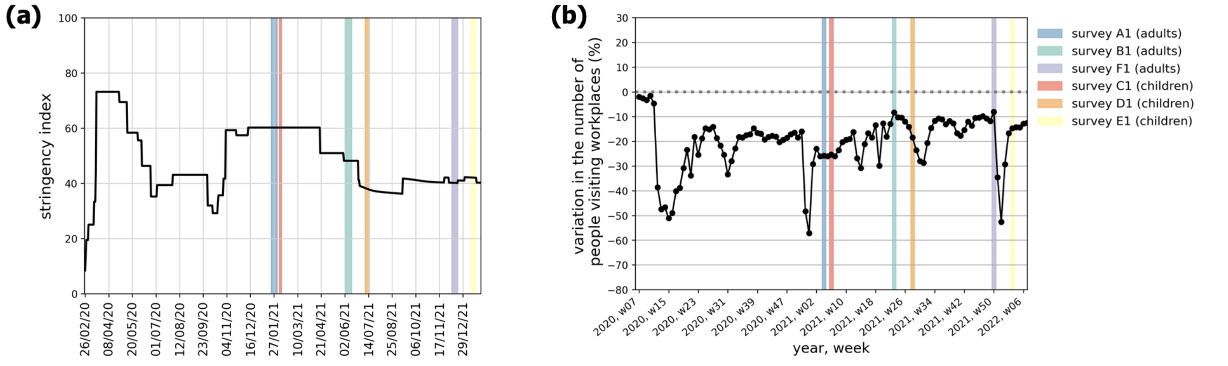

Figure S1. Pandemic context of the social contact surveys in Switzerland. (a) Sringency Index (Oxford COVID-19 Government Response Tracker[3]). **(b)** Weekly mobility variation related to workplaces (Google mobility data[4]). In both panels, colored shaded areas refer to the period of data collection of the contact surveys.

1.2 Weighted SEP index

The SEP index is defined for each residential building in Switzerland. Participants in the contact survey did not declare their address but only their municipality of residence. Therefore, to assign a

SEP index to each participants, we computed a weighted average of the SEP index wS_A by municipality A as

$$wS_A = \sum_{h \in A} \frac{n_h S_h}{\sum_{h \in A} n_h}$$

where h is any residential building in municipality A, n_h is the number of people living in residential building h, and S_h is the corresponding SEP index. In other words, we mapped the distribution of SEP values at residential building level to a distribution at individual level, and then computed the average SEP per individual living in the municipality. To inform n_h, we used the population and household statistic (STATPOP) 2021-2022 dataset[5]. We then used the median value of the weighted average SEP by municipality to classify participants in a given municipality as low or high SEP. Results are displayed in **Fig. S2**.

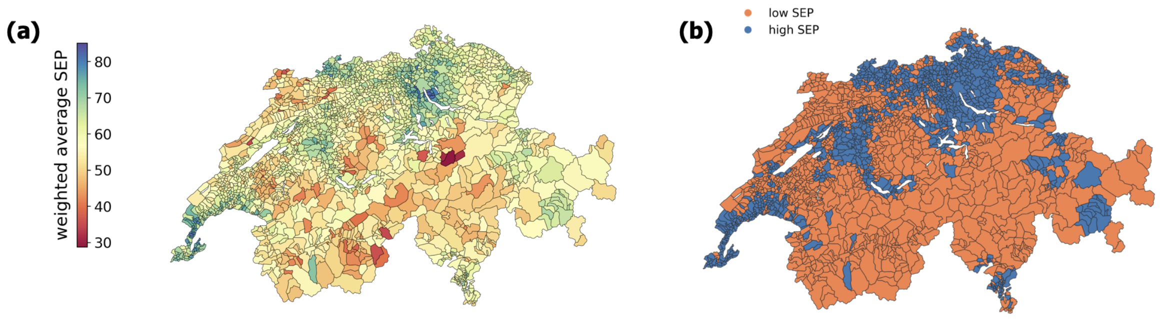

Figure S2. Weighted SEP index by municipality. (a) Map of municipalities in Switzerland color-coded based on the value of the weighted SEP. **(b)** Map of municipalities in Switzerland classified as low SEP (orange) or high SEP (blue) using the median weighted SEP as a threshold.

1.3 Population size by age group, SEP level and education level

Participants in the CoMix survey could be directly classified in terms of age group, SEP level (based on the municipality of residence) and education level based on the information available (see section above). For the general Swiss population, we extracted an estimate of population sizes using the following procedure.

We used data by education level (according to the 3-group classification used by the Swiss Education System, i.e., without post-compulsory education, upper-secondary level, tertiary level) available for each district (an administrative area coarser than municipalities) for the permanent adult population (above 25 y.o.). For each district, we computed the population profile by education level, and we assigned the weighted SEP index of the district (computed analogously to the weighted SEP index by municipality). For each district, we also computed the population size of the four age groups of interest (0-14, 15-24, 25-64, 65+ y.o.). To infer the missing interaction between age and education level in each district, we projected the distribution by education level and age observed at national level to the sizes of the groups by age and by education level for each district.

Quotas in the recruitment of survey participants for the CoMix were set on age and sex, allowing for a representative distribution in the sample along these variables, but not on education level and SEP. In **Fig. S3** we checked for the representativeness of the sample including education level and SEP. We found good agreement between the survey sample and the general population.

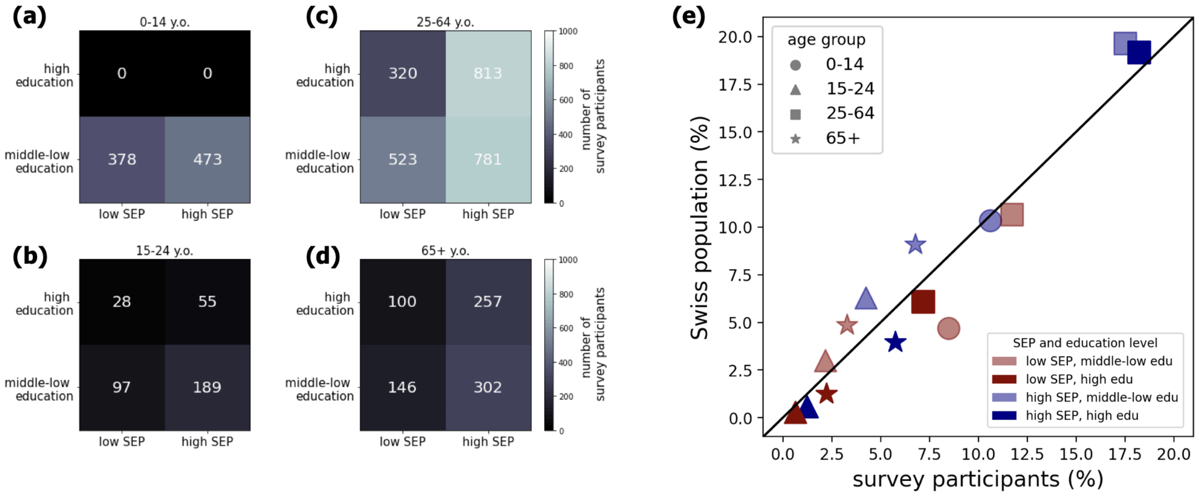

Figure S3. Representativeness of survey population. (a) Number of participants 0-14 y.o. divided by education level (y axis) and SEP level (x axis). **(b-d)** As in panel (a), showing the number of participants in age groups 15-24, 25-64 and 65+ y.o., respectively.**(e)** Population profile by age, SEP and SES, in Switzerland (y axis) and in the survey (x axis). Color of the symbols indicates the SEP and education level; the symbol type indicates the age group.

2. Supplementary Results: contact determinants

2.1 Contact activity by survey wave

For completeness, we report in this section the results analogous to **Fig. 2a-d** in the main text, stratified by survey wave (**Fig. S4**).

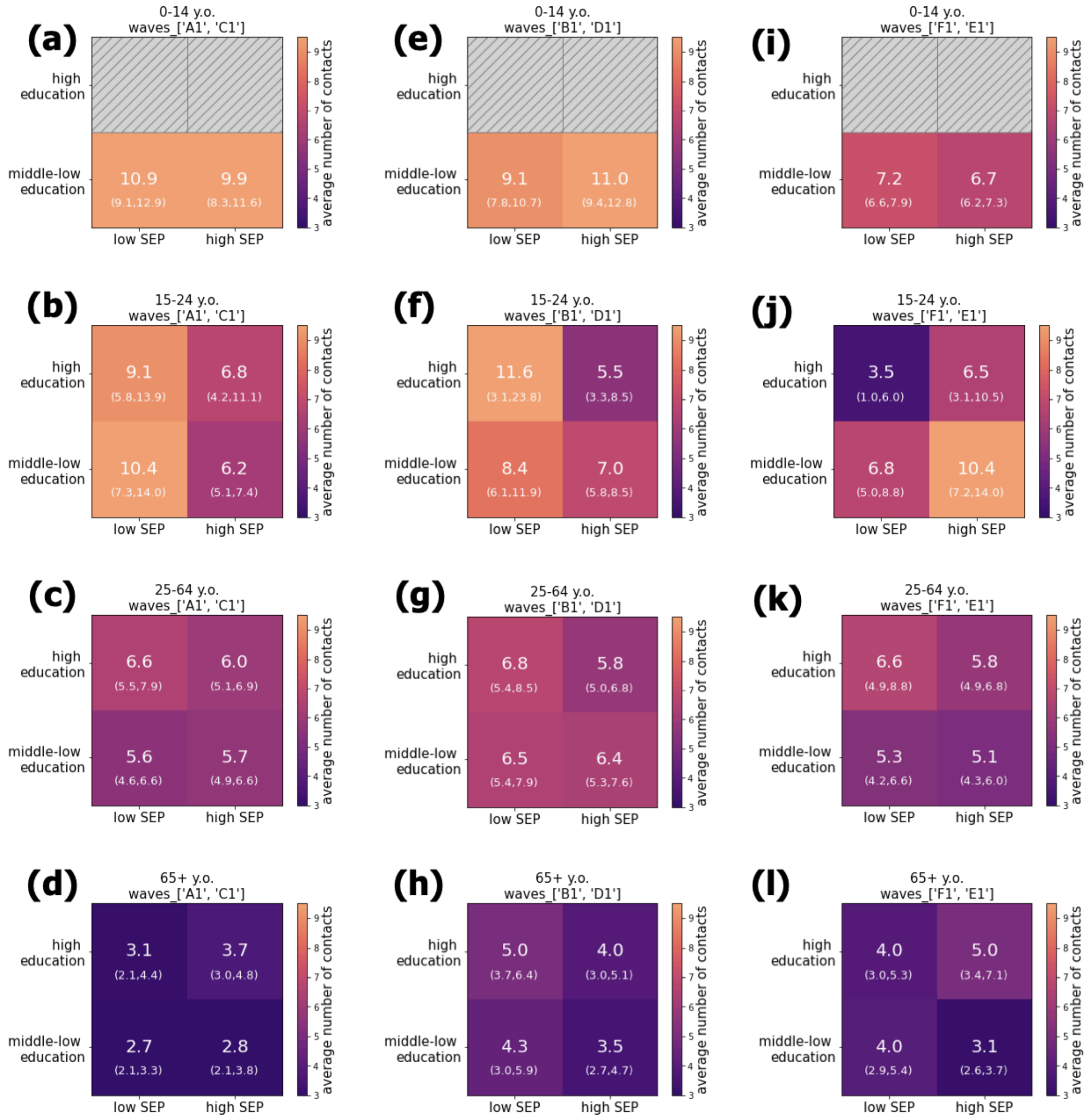

Figure S4. Contact activity by socioeconomic group. (a-d) Average number of contacts for each individual-based (education level) and area-based (SEP) socio-economic group. The heatmap shows the crude mean number of contacts (along with 95% confidence interval obtained with 1000 bootstraps) engaged by a participant belonging to one of the four socio-economic groups, depending on the age group **(a)** 0-14 y.o. (children), **(b)** 15-24 y.o. (young adults) **(c)** 25-64 y.o. (adults) and **(d)** 65+ y.o. (seniors). Results refer to survey waves A1 and C1 (January - February 2021). **(e-h)** As in panels (a-d), results refer to survey waves B1 and D1 (June - July 2021). **(i-l)** As in panels (a-d), results refer to survey waves F1 and E1 (December 2021 - January 2021).

2.2 Regression model: sensitivity analyses

In this section, we present the results of some model variations in the regression analysis. In particular, we tested the inclusion of an additional explanatory variable (population density) and a different definition of the outcome variable, where we looked specifically at contacts at work and contacts outside the household rather than contacts overall. For contacts outside the household, we tested two definitions: contacts with a known location different from 'home' or 'household' (excluding contacts with missing location), or any contact outside home (including contacts with missing location). We also report results of the sensitivity analysis adding an interaction term with age for the household income variable, and distinguishing by survey wave.

For what concerns contacts at work, the number of contacts was very low for all age groups except 25-64 y.o. (as expected). Therefore, the estimated rate ratios were either non significant or highly uncertain. For adults in 25-64 y.o., we found a negative effect of high SEP and high education level on the number of contacts at work, but the effect was very small (**Fig. S5a**).

For contacts outside home (in both definitions excluding or including unspecified locations, **Fig. S5b,c**), we found again a positive effect of high education level on the number of contacts in seniors (65+ y.o.) and a negative effect of SEP on the number of contacts in adults (25-64 y.o.), consistently to what was found in the main analysis on overall contacts.

We computed the population density (by km2) as the population divided by the area (km2). The area was retrieved by the polygons in the shapefile of Switzerland[6], using an appropriate projection (Swiss Oblique Mercator, EPSG:2056). However, this measure does not account for large non-inhabited areas in Switzerland (due to e.g. mountains), especially in the south of the country. This is visible in the map shown in Ref.[7]. This means that a municipality which overlaps with a mountain area may have a low population density which is not representative of the effective population density in the inhabited areas. To correct for this bias, we computed the effective population density, as a weighted mean of the population by hectare. We used publicly available data on the population by hectare[8]. We included the effective population density as a variable in the regression model, categorized as 'low', 'baseline' or 'high', using quartiles at 25% and 75% as threshold. We found that the estimates of the rate ratio did not change significantly. Results are shown in **Fig. S6b**.

Stratifying by survey wave, we found that the main results remained consistent across waves: seniors with higher education level reported more contacts with respect to middle-low education, and adults living in high SEP areas reported less contacts with respect to low SEP areas (**Fig. S7**).

In the main analysis, we found that household income was also positively associated with the number of contacts. This holds for all age groups after inclusion of an interaction term with age. The effect is smaller in adults and larger in young adults and seniors (**Fig. S8b**).

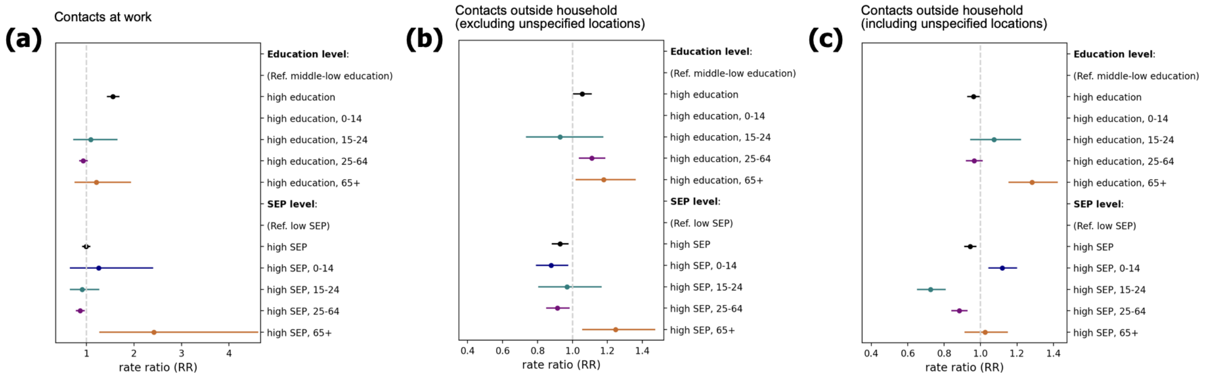

Figure S5. Changing the type of contacts in the outcome variable. (a) Same analysis as the one shown in **Fig. 2e** in the main text, but using the number of contacts at work as the outcome variable. **(b)** As in panel (a), but using the number of contacts outside home (excluding unspecified locations) as outcome variable. **(c)** As in panel (a), but using the number of contacts outside home (including unspecified locations) as outcome variable. Black and colored indicate the rate ratio (RR) in the univariate and multivariate regression model with interaction term with age, respectively. Bars indicate 95% confidence intervals.

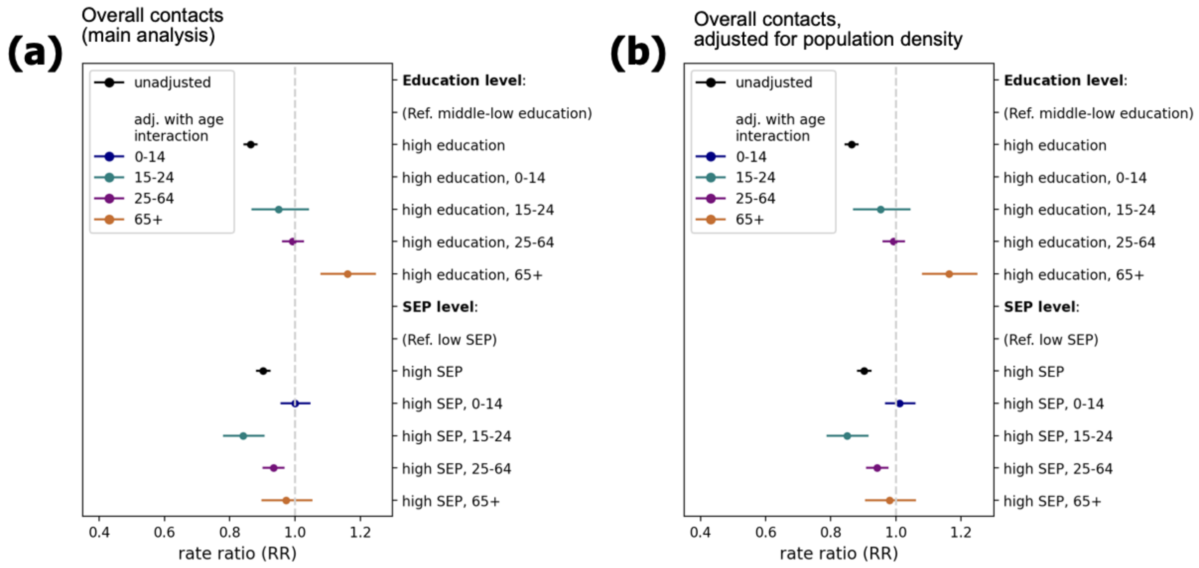

Figure S6. Adding population density as an explanatory variable. (a) Same panel as the one shown in **Fig. 2e** in the main text. Black and colored indicate the rate ratio (RR) in the univariate and multivariate regression model with interaction term with age, respectively. Bars indicate 95% confidence intervals. **(b)** As in panel (a), but adding the effective population density as a variable in the regression model.

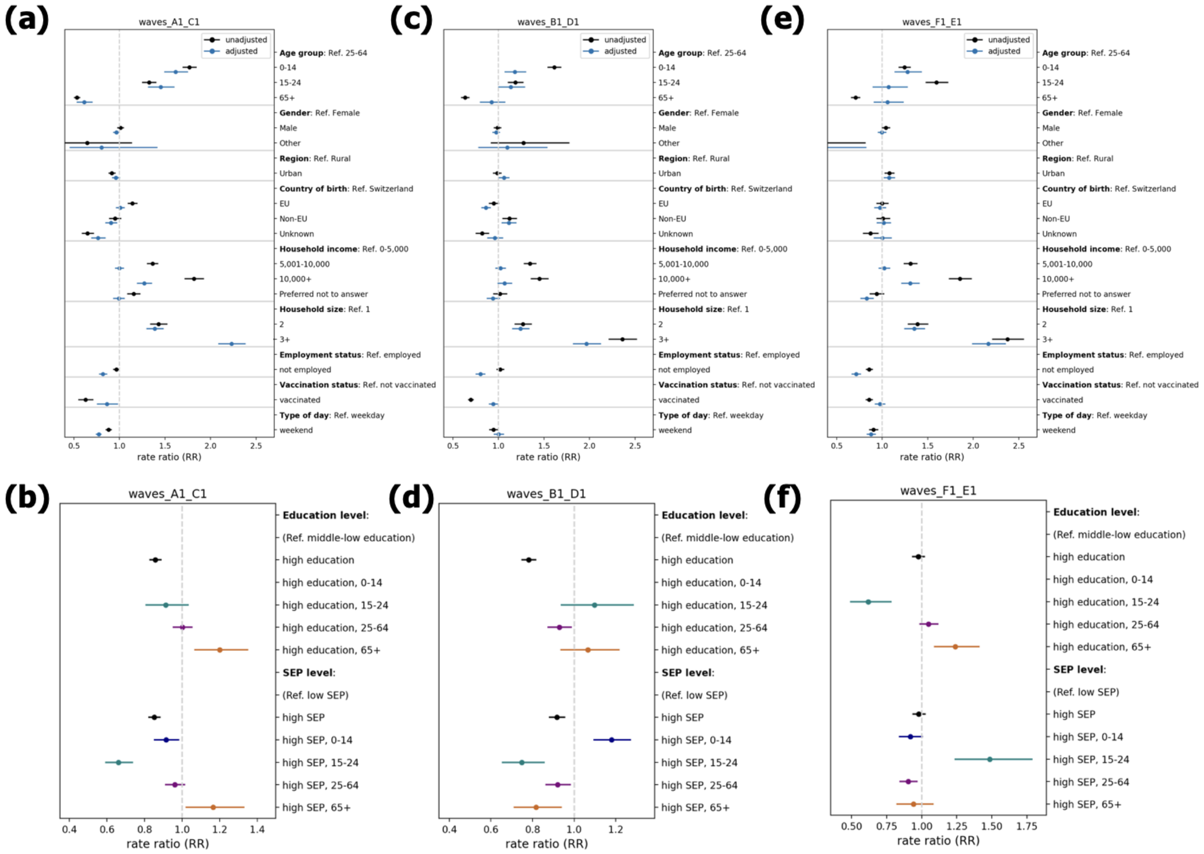

Figure S7. Stratifying by survey wave. (a-b) Same panels as the ones shown in **Fig. 2e,f** in the main text, however considering only participants in the first survey period (January and February 2021 for adults and children respectively). **(c-d)** As in panels (a-b), but considering only participants in the second survey period (June and July 2021). **(e-f)** As in panels (a-b), but considering only participants in the third survey period (December 2021 and January 2022). Black and colored indicate the rate ratio (RR) in the univariate and multivariate regression model with interaction term with age, respectively. Bars indicate 95% confidence intervals.

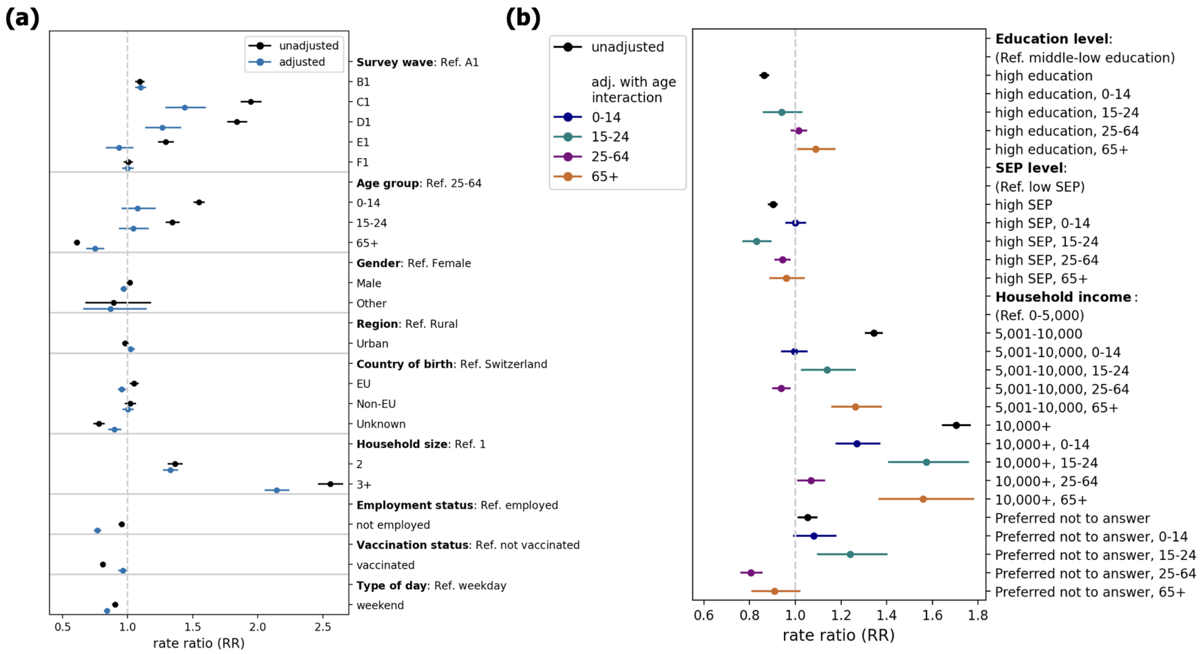

Figure S8. Adding an interaction term between age and income. (a-b) Same panels as the ones shown in **Fig. 2e,f** in the main text, but including an interaction term not only for SEP and education level but also for household income. Black and colored indicate the rate ratio (RR) in the univariate and multivariate regression model with interaction term with age, respectively. Bars indicate 95% confidence intervals.

3. Supplementary Methods: construction of contact matrices

3.1 Reciprocity correction

In this section, we follow the notation introduced in the main text. We want to apply a reciprocity correction to the rectangular matrix $\overline{M}_{(i,v,d),j}$, whose elements represent the number of contacts that a participant in age group i, SEP level v and education level d has with individuals in age group j. We applied a similar method as the one introduced in Ref.[9]. For each couple of age groups i and j, we computed $B^{i,j}_{v,d} = N_{(i,v,d)}\overline{M}_{(i,v,d),j}/N_i M_{ij}$, i.e the proportion of contacts engaged by individuals in socio-economic group (v, d) out of the total number of contacts between age group i and age group j. We then applied these proportions to the elements of the age-stratified reciprocal matrix $M^{rec}_{i,j}$, to obtain $\overline{M}^{rec}_{(i,v,d),j} = B^{i,j}_{v,d} M^{rec}_{i,j}$, so that $\sum_{v,d} N_{(i,v,d)} \overline{M}^{rec}_{(i,v,d),j} / N_i = M^{rec}_{i,j}$. In other words, this correction ensures that, when aggregating the matrix $\overline{M}^{rec}_{(i,v,d),j}$ over the socio-economic groups (v, d), we can retrieve the reciprocal age-stratified matrix $M^{rec}_{i,j}$.

3.2 Matrix expansion

We expanded the adjusted intermediate matrix $\overline{M}^{rec}$ (**Fig. S9a**) to a fully stratified contact matrix $\widehat{M}_{s,t}$ (**Fig. S9b**). To derive the elements of the matrix $\widehat{M}$, we shall solve a system of linear equations, with constraints due to the properties of the structure of the social contact matrix. In particular, the matrix $\widehat{M}$ is required to fulfill conditions on (i) reciprocity, i.e., the total number of contacts $N_s \widehat{M}_{s,t}$ between individuals in group s and group t must be equal to $N_t \widehat{M}_{t,s}$; (ii) aggregation, i.e., the number of contacts summed over all socio-economic groups (excluding age) must be consistent with the elements of the intermediate contact matrix $\overline{M}^{rec}$, so that $\sum_{u,c} \widehat{M}_{(i,v,d),(j,u,c)} = \overline{M}^{rec}_{(i,v,d),j}$; (iii) positivity, i.e., the number of contacts in each cell cannot be negative.

Practically, the global system can be solved through 10 independent linear systems corresponding to specific blocks of the contact matrix: 4 diagonal 4x4 blocks (one for each age group) with 16 variables, and 6 couples of off-diagonal 4x4 blocks, for a total of 32 variables, corresponding to couples of interacting age groups (i, j) with $i \neq j$. In order to solve the system for each diagonal block (mixing within an age group), it is required to set 6 free parameters, while the remaining 10 are derived from the conditions on reciprocity and aggregation. We defined 6 parameters $q \in (0, 1)$, that can be interpreted as assortativity parameters along the education level dimension and the SEP dimension. To solve the system for the off-diagonal blocks (mixing across age groups), we found that it is required to set 9 free parameters, while the remaining 23 are derived from reciprocity and aggregation. Similarly to above, we defined 9 parameters describing the assortativity and the general distribution of contacts along the education level and SEP dimension. In total, to derive the expanded contact matrix, it is required to set the value of 55 free parameters. In **Table S1**, we report the values of the 55 parameters chosen for the matrix displayed in **Fig. 3c** (here also reported in **Fig. S9b**).

We explored the parameter space through random sampling in the range $(0, 1)$ for each q, and selected combinations of parameter values that ensure the positivity of each block, and therefore of the expanded contact matrix. We exploited some analytical conditions on some parameters to restrict the parameter space and optimize the search. Additional details are contained in the following two sections.

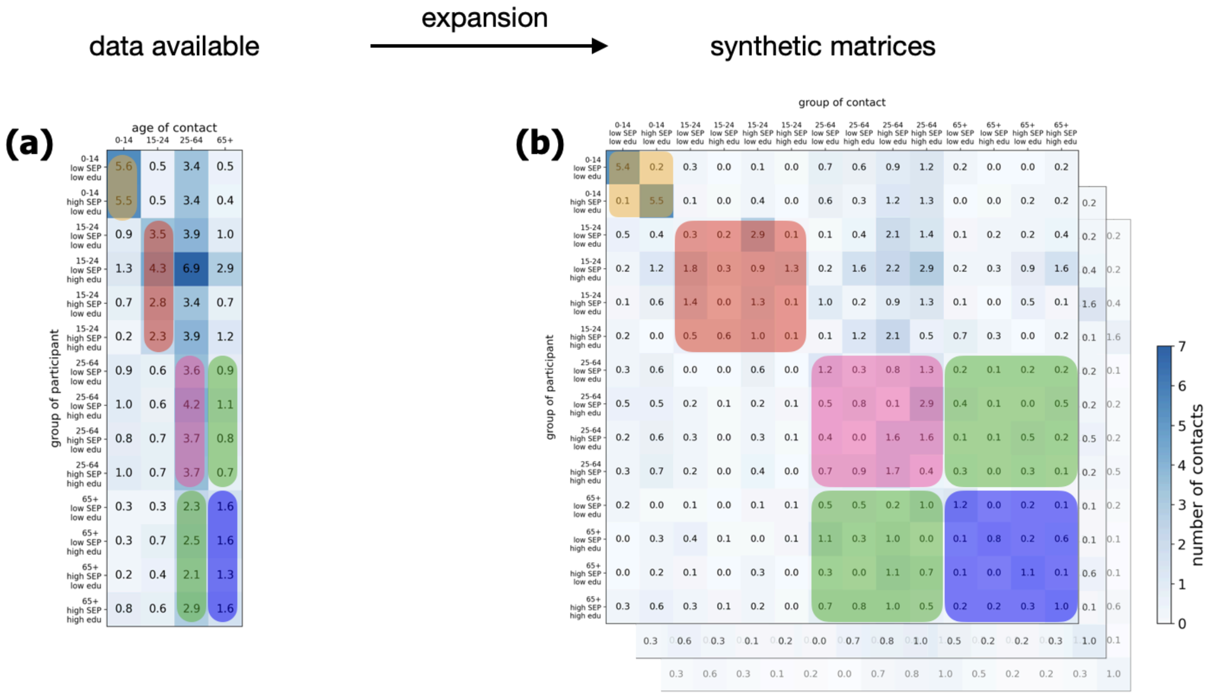

Figure S9. Synthetic expansion. (a) Intermediate contact matrix. **(b)** Example of expanded contact matrix. The figure illustrates the correspondence between the elements in the intermediate matrix (panel (a)) and the elements of the fully stratified matrix (panel (b), one example of the set of possible matrices). Some blocks are highlighted in color for illustrative purposes. The 4x4 diagonal block in pink represents the mixing within the age group of adults (25-64 y.o.), across the 4 socio-economic groups (low/high SEP, middle-low/high education), which must be compatible under aggregation (sum per row) with the elements in pink in the intermediate matrix. The off-diagonal block in green represents the mixing between adults (25-64 y.o.) and seniors (65+ y.o.), compatible under aggregation with the elements in green in the intermediate matrix. The matrix in panel (b) is built in order to satisfy reciprocity, i.e., $N_s M_{s,t} = N_t M_{t,s}$ for any group s, t (3-tuple of age group i, SEP level v and education level d).

Mixing within an age group (diagonal blocks)						
Age group i	$q^{i}_{v_1,d_1}$	$q^{i}_{v_1,d_2}$	$q^{i}_{v_2,d_1}$	$q^{i}_{v_2,d_2}$	$q^{i}_{v_1}$	$q^{i}_{d_1}$
Children	////////	////////	////////	////////	0.97	////////
Young adults	0.09	0.08	0.45	0.06	0.17	0.94
Adults	0.34	0.19	0.44	0.11	0.37	0.56
Seniors	0.79	0.50	0.80	0.62	0.74	0.90

Mixing across two age groups (off-diagonal blocks)									
Age groups i, j	$q^{i,j}_{v_1,d_1}$	$q^{i,j}_{v_1,d_2}$	$q^{i,j}_{v_2,d_1}$	$q^{i,j}_{v_2,d_2}$	$q^{i,j}_{v_1}$	$q^{i,j}_{d_1}$	$r^{i,j}_{1}$	$r^{i,j}_{2}$	$r^{i,j}_{3}$
Children & young adults	0.65	////////	0.71	////////	0.67	////////	////////	////////	////////
Children & adults	0.20	////////	0.35	////////	0.37	////////	////////	////////	////////
Children & seniors	0.51	////////	0.36	////////	0.52	////////	////////	////////	////////
Young adults & adults	0.02	0.23	0.26	0.12	0.15	0.59	0.11	0.41	0.51
Young adults & seniors	0.11	0.12	0.69	0.16	0.28	0.65	0.22	0.33	0.52
Adults & seniors	0.29	0.06	0.63	0.14	0.46	0.64	0.22	0.04	0.63

Table S1. Parameter values. List of parameter values used to generate the example of an expanded synthetic matrix shown in **Fig. 3c** (and **Fig. S9b**). For the definition of the parameters, see the sections below. The indexes v_1 and v_2 refer to the two SEP levels (low SEP and high SEP, respectively). The indexes d_1 and d_2 refer to the two education levels (middle-low and and high education level, respectively). For children, the assortativity parameters along the education level dimension are not defined because all children are classified as middle-low education.

3.2.1 Inferring matrix elements in the diagonal blocks

For each of the four diagonal blocks (one for each age group), composed of 16 unknowns, we can write down 10 conditions, namely 4 conditions for the aggregation and 6 conditions on the reciprocity. We then defined 6 conditions based on 6 free parameters that describe how contacts within the same age group are distributed across different SEP and education levels. More specifically, we defined one assortativity parameter for each of the 4 socio-economic groups (couple (v, d) of SEP and education level), one assortativity parameter in the SEP dimension only, and one assortativity parameter in the education level dimension only.

Given one age group i, we can define four parameters $q^{i}_{v,d} \in (0, 1)$ for any couple of SEP level $v \in \{v_1, v_2\}$ and education level $d \in \{d_1, d_2\}$ as the proportion of contacts engaged within the same group, i.e., $q^{i}_{v,d} = \widehat{M}_{(i,v,d),(i,v,d)} / \sum_{u,c} \widehat{M}_{(i,v,d),(i,u,c)}$, where $\widehat{M}_{(i,v,d),(i,v,d)}$ are the diagonal elements of the matrix $\widehat{M}$.

We can then define the assortativity parameter along the SEP dimension and education level dimension separately by looking at the aggregated squared matrices $\widehat{M}_{(i,v),(i,u)}$ and $\widehat{M}_{(i,d),(i,c)}$ expanded only on the SEP level and age, or education level and age, respectively, and the corresponding rectangular matrices $\overline{M}_{(i,v),j}$ and $\overline{M}_{(i,d),j}$ aggregated over the socio-economic group of the contactee. In the education level dimension, let us call d_1 the group with middle-low education, and d_2 the group with high education. We can define $q^i_{d_1} \in (0,1)$ as

$$q^i_{d_1} = \widehat{M}_{(i,d_1),(i,d_1)} / (\widehat{M}_{(i,d_1),(i,d_1)} + \widehat{M}_{(i,d_1),(i,d_2)}) = \widehat{M}_{(i,d_1),(i,d_1)} / \overline{M}_{(i,d_1),i}$$

i.e., the share of contacts that one individual in education level d_1 and age group i engages with the same group, regardless of the SEP level. Analogously, we can define $q^i_{v_1} \in (0,1)$ using v_1 and v_2 for the low SEP and high SEP groups.

For each age group i, we can then numerically solve the system of 16 equations for any combination of values for the six free parameters ($q^i_{v_1,d_1}$, $q^i_{v_1,d_2}$, $q^i_{v_2,d_1}$, $q^i_{v_2,d_2}$, $q^i_{v_1}$, $q^i_{d_1}$). In principle, any assortativity parameter q defined above could take any value between 0 and 1, by construction. However, not all values would be compatible with the condition of positivity, which we have not used so far.

For example, for $q^i_{d_1}$ we can write down the following system

$$\widehat{M}_{(i,d_1),(i,d_1)} + \widehat{M}_{(i,d_1),(i,d_2)} = \overline{M}_{(i,d_1),i}$$

$$\widehat{M}_{(i,d_2),(i,d_1)} + \widehat{M}_{(i,d_2),(i,d_2)} = \overline{M}_{(i,d_2),i}$$

$$\widehat{M}_{(i,d_1),(i,d_2)} N_{(i,d_1)} = \widehat{M}_{(i,d_2),(i,d_1)} N_{(i,d_2)}$$

$$q^i_{d_1} = \widehat{M}_{(i,d_1),(i,d_1)} / \overline{M}_{(i,d_1),i}$$

where $\widehat{M}_{(i,d),(i,c)}$ with $d, c \in \{d_1, d_2\}$ are the four unknown variables, while $\overline{M}_{(i,d),i}$ and $N_{(i,d)}$ are known from the data. The first two conditions satisfy the aggregation property, while the third ensures reciprocity. By analytically solving this system, we can derive the following condition on $q^i_{d_1}$:

$$\widehat{M}_{(i,d_2),(i,d_2)} = \overline{M}_{(i,d_2),i} - (N_{(i,d_1)} / N_{(i,d_2)}) \overline{M}_{(i,d_1),i} (1 - q^i_{d_1}) \geq 0$$

$$\Rightarrow q^i_{d_1} \geq 1 - (\overline{M}_{(i,d_2),i} N_{(i,d_2)}) / (\overline{M}_{(i,d_1),i} N_{(i,d_1)})$$

This condition is necessary in order for $\widehat{M}_{(i,d_2),(i,d_2)}$ to be non-negative.

We can derive an analogous condition for $q^i_{v_1}$ by looking at the 2x2 diagonal blocks of the matrix $\widehat{M}_{(i,v),(i,u)}$. Hence, when exploring the parameter space ($q^i_{v_1,d_1}$, $q^i_{v_1,d_2}$, $q^i_{v_2,d_1}$, $q^i_{v_2,d_2}$, $q^i_{v_1}$, $q^i_{d_1}$), we can restrict the values for $q^i_{v_1}$, $q^i_{d_1}$ to the ones displayed in **Table S2**.

	SEP	Education level
Children (0-14 y.o.)	$0 \le q^{(0-14)}_{v_1} \le 1$	$q^{(0-14)}_{d_1} = 1$
Young adults (15-24 y.o.)	$0 \le q^{(15-24)}_{v_1} \le 1$	$0.90 \le q^{(15-24)}_{d_1} \le 1$
Adults (25-64 y.o.)	$0 \le q^{(25-64)}_{v_1} \le 1$	$0.13 \le q^{(25-64)}_{d_1} \le 1$
Seniors (65+ y.o.)	$0 \le q^{(65+)}_{v_1} \le 1$	$0.55 \le q^{(65+)}_{d_1} \le 1$

Table S2. Assortativity conditions for diagonal blocks. Conditions on assortativity in the education level or SEP dimension necessary for positivity of the matrix elements in the four 4x4 diagonal blocks. The parameter $q^{(0-14)}_{d_1}$ is equal to 1, because all children are classified as middle-low education, therefore all contacts are engaged within this group.

We can interpret the values in the table as follows. For example, the condition $q^{(15-24)}_{d_1} \ge 0.9$ means that, out of the number of contacts that an individual with age (15-24 y.o.) and middle-low education level has on average with people in the (15-24 y.o.) group, at least 90% must be shared with people with the same middle-low education level.

We could in principle derive some conditions on the parameters $q^i_{v,d}$ as well, however that would entail to analytically solve the system in 16 variables, and would require more cumbersome computations. Hence, we will check for values ranging between 0 and 1 through random sampling from a uniform distribution. In practice, we applied the following procedure. After a preliminary exploration, we identified some empirical constraints, i.e., some threshold values limiting the observed distribution. We used them in a second round of exploration, where we tested 2,000,000 parameter combinations by sampling each parameter from a uniform distribution over the corresponding interval, either (0-1) or a restricted interval based on the empirical or analytical constraints. We selected only those combinations leading to a positive contact matrix. The resulting selected distributions are illustrated in **Fig. S10**. For each parameter, we show the median value and the value expected from proportional mixing. We can see that in the majority of cases matrices display assortativity values higher than the ones expected from homogeneous mixing.

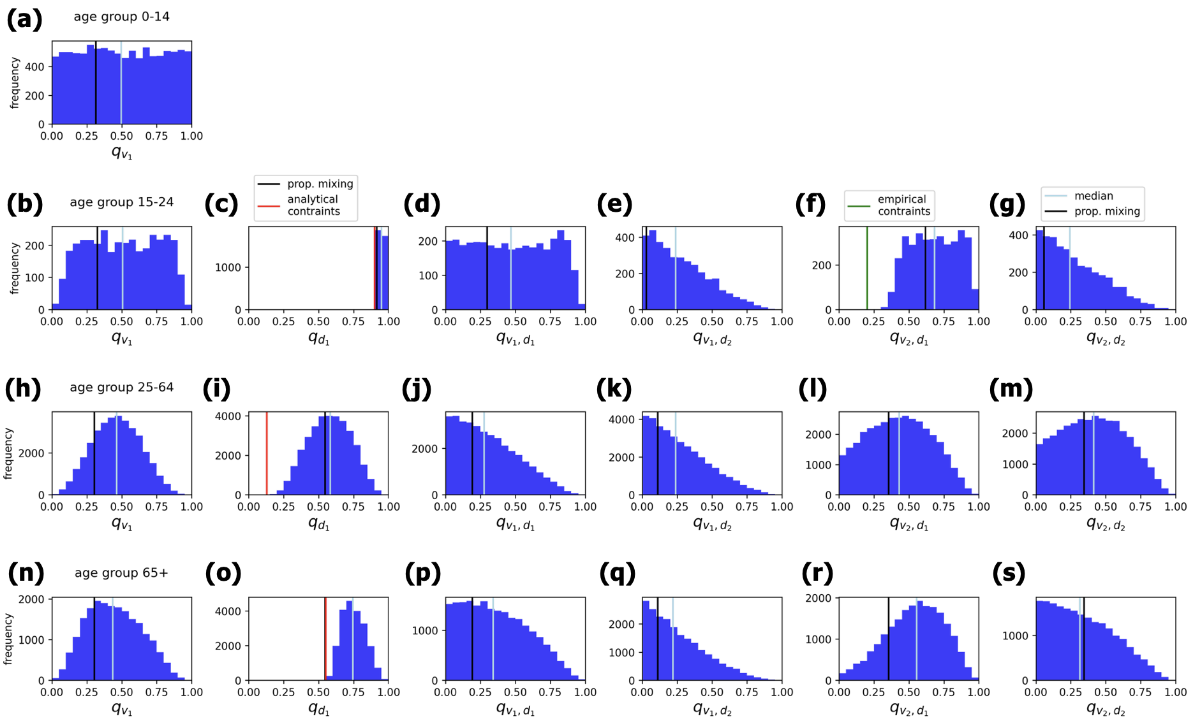

Figure S10. Parameter distributions for diagonal blocks. (a) For children in 0-14 y.o., the system of equations is reduced because all children belong to the middle-low education group, therefore, only one parameter of assortativity $q_{v_1}^{(0-14)}$ is needed. **(b-g)** For young adults 15-24 y.o., we show the frequency of values of the 6 free parameters (columns) of assortativity which allow solving the system and derive a positive matrix. **(h-m)** Same as panels (b-g), but for adults 25-64 y.o. **(n-s)** Same as panels (b-g), but for seniors 65+ y.o. In each panel we show the median of the observed distribution (vertical line in light blue), and the value expected from homogeneous mixing, i.e., mixing proportional to the size of the subgroups (vertical black line).

3.2.2 Inferring matrix elements in the off-diagonal blocks

For each of the six coupled off-diagonal blocks with 32 variables, corresponding to a couple of age groups i and j with $i \neq j$, we can write 16 conditions on reciprocity, and 8 conditions on the aggregation. However, it turns out that one of them is linearly dependent on the others, so we can drop one condition on reciprocity and then set 9 remaining conditions in order to solve the system. Similarly to what was done before, we can define 9 free parameters which describe how contacts are distributed along the SEP and education level dimensions. We choose to fix: (i) 4 conditions on the assortativity on the (sub)diagonal elements; (ii) 2 conditions on the assortativity in the education level and SEP dimension separately, similarly to what was done above; (iii) 3 remaining parameters with no particular link with assortativity, defined on the share of contacts in one element outside of the sub-diagonal elements. More precisely, for a couple of age groups i and j:

(i) $$q_{v,d}^{i,j} = \widehat{M}_{(i,v,d),(j,v,d)} / \sum_{u,c} \widehat{M}_{(i,v,d),(j,u,c)} \text{ for any } v \in \{v_1, v_2\},\ d \in \{d_1, d_2\}$$

(ii)a $$q_{d_1}^{i,j} = \widehat{M}_{(i,d_1),(j,d_1)} / (\widehat{M}_{(i,d_1),(j,d_1)} + \widehat{M}_{(i,d_1),(j,d_2)}) = \widehat{M}_{(i,d_1),(j,d_1)} / \overline{M}_{(i,d_1),j}$$

(ii)b $q^{i,j}_{v_1} = \widehat{M}_{(i,v_1),(j,v_1)} / (\widehat{M}_{(i,v_1),(j,v_1)} + \widehat{M}_{(i,v_1),(j,v_2)}) = \widehat{M}_{(i,v_1),(j,v_1)} / \overline{M}_{(i,v_1),i}$

(iii)a $r^{i,j}_{1} = \widehat{M}_{(i,v_1,d_1),(j,v_1,d_2)} \,/\, \sum_{u,c} \widehat{M}_{(i,v_1,d_1),(j,u,c)}$

(iii)b $r^{i,j}_{2} = \widehat{M}_{(i,v_1,d_2),(j,v_2,d_1)} \,/\, \sum_{u,c} \widehat{M}_{(i,v_1,d_2),(j,u,c)}$

(iii)c $r^{i,j}_{3} = \widehat{M}_{(i,v_2,d_1),(j,v_2,d_2)} \,/\, \sum_{u,c} \widehat{M}_{(i,v_2,d_1),(j,u,c)}$

Similarly to what was done above, we can analytically solve the system for the blocks of the contact matrix expanded only on the education level and age, or only on the SEP level and age. We find that a necessary condition for the positivity is:

$$1 - (\overline{M}_{(j,d_2),i} N_{(j,d_2)})/(\overline{M}_{(i,d_1),j} N_{(i,d_1)}) \le q^{i,j}_{d_1} \le 1 - (\overline{M}_{(j,d_2),i} N_{(j,d_2)})/(\overline{M}_{(i,d_1),j} N_{(i,d_1)}) + (\overline{M}_{(i,d_2),j} N_{(i,d_2)})/(\overline{M}_{(i,d_1),j} N_{(i,d_1)})$$

and an analogous condition can be written for $q^{i,j}_{v_1}$. Hence, when exploring the parameter space $(q^{i,j}_{v_1,d_1}, q^{i,j}_{v_1,d_2}, q^{i,j}_{v_2,d_1}, q^{i,j}_{v_2,d_2}, q^{i,j}_{v_1}, q^{i,j}_{d_1}, r^{i,j}_{1}, r^{i,j}_{2}, r^{i,j}_{3})$, we can restrict the values for $q^{i,j}_{v_1}$, $q^{i,j}_{d_1}$ to the ones displayed in **Table S3**.

	SEP	Education level
Young adults & adults	$0 \le q^{(15-24),(25-64)}_{v_1} \le 0.77$	$0.49 \le q^{(15-24),(25-64)}_{d_1} \le 0.62$
Young adults & seniors	$0 \le q^{(15-24),(65+)}_{v_1} \le 0.62$	$0.53 \le q^{(15-24),(65+)}_{d_1} \le 0.75$
Adults & seniors	$0 \le q^{(25-64),(65+)}_{v_1} \le 0.94$	$0.43 \le q^{(25-64),(65+)}_{d_1} \le 1$
Children & young adults	$0 \le q^{(0-14),(15-24)}_{v_1} \le 1$	$0.93 \le q^{(0-14),(15-24)}_{d_1} \le 1$
Children & adults	$0 \le q^{(0-14),(25-64)}_{v_1} \le 0.99$	$0.51 \le q^{(0-14),(25-64)}_{d_1} \le 1$
Children & seniors	$0 \le q^{(0-14),(65\ )}_{v_1} \le 0.76$	$0.44 \le q^{(0-14),(65+)}_{d_1} \le 1$

Table S3. Assortativity conditions for off-diagonal blocks. Conditions on assortativity in the education level or SEP dimension necessary for positivity of the matrix elements in the six off-diagonal blocks.

We can interpret the values in the table as follows. For example, the condition $q^{(15-24),(65+)}_{v_1} \le 0.62$ means that at maximum 62% of the number of contacts engaged by an individual with age (15-24 y.o.) and low SEP level with 65+ y.o. is shared with people with the same low SEP level.

Similarly to what was done for the diagonal blocks, we explored the 9-dimensional parameter space through random sampling. In practice, we applied the following procedure. After a preliminary exploration, we identified some empirical constraints, i.e., some threshold values limiting the observed

distribution. We used them in a second round of exploration, where we tested 2,000,000 parameter combinations by sampling each parameter from a uniform distribution over the corresponding interval, either (0-1) or a restricted interval based on the empirical or analytical constraints.

We selected only those combinations leading to a positive contact matrix. The resulting selected distributions are illustrated in **Fig. S11**. For children in (0-14), the system is reduced to 8 equations because they all belong to the group with middle-low education, therefore the number of free parameters needed is 3 rather than 9. They are shown in **Fig. S12**.

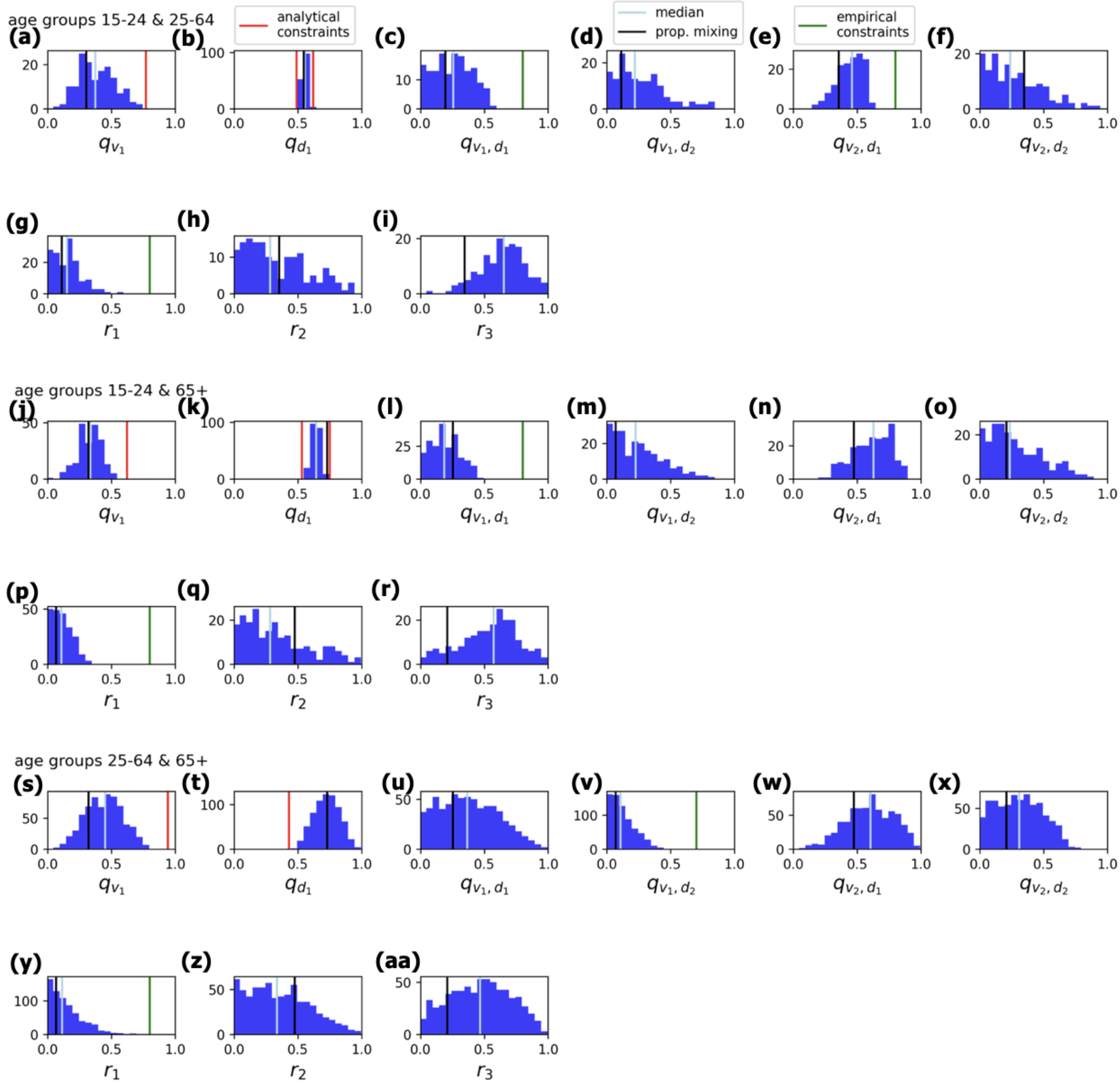

Figure S11. Parameter distributions for off-diagonal blocks (ecluding children). (a-i) Frequency of values of the 9 free parameters in the selected combinations, for the interaction young adults - adults. **(j-r)** Frequency of values of the 9 free parameters in the selected combinations, for the interaction young adults - seniors. **(s-aa)** Frequency of values of the 9 free parameters in the selected combinations, for the interaction adults - seniors. In each panel we show the median of the observed distribution (vertical line in light blue), and the value expected from homogeneous mixing, i.e., mixing proportional to the size of the subgroups (vertical black line).

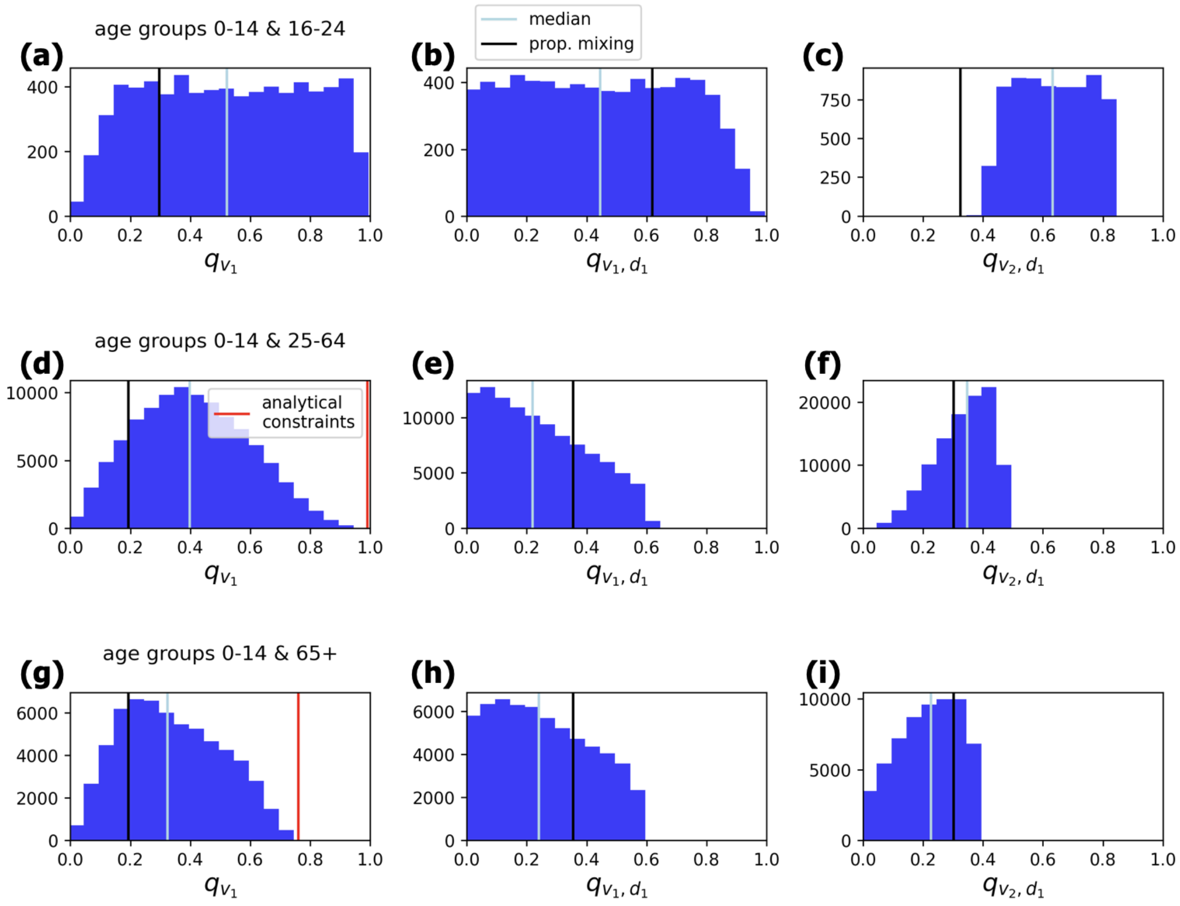

Figure S12. Parameter distributions for off-diagonal blocks involving children. (a-c) Frequency of values of the 3 free parameters, for the interaction children - young adults. **(d-f)** Frequency of values of the 3 free parameters, for the interaction children - adults. **(g-i)** Frequency of values of the 3 free parameters, for the interaction children - seniors. In each panel we show the median of the observed distribution (vertical line in light blue), and the value expected from homogeneous mixing, i.e., mixing proportional to the size of the subgroups (vertical black line).

3.3 Assortativity index

In the previous section, we defined assortativity parameters specific for each age group and socio-economic group, required to derive the fully stratified contact matrix. Here, we summarise the characteristics of the matrix by defining a single assortativity index (one for education level, and one for SEP), aggregating over age groups and over one of the two social dimensions.

Let us consider the expanded contact matrix $\widehat{M}_{(i,v,d),(j,u,c)}$. We can aggregate the matrix over the age and education level dimensions, in order to obtain the 2x2 matrix $\widehat{M}_{v,u}$, by summing the columns u, c and computing a weighted mean on the rows i, j as follows:

$$\widehat{M}_{v,u} = \sum_{d,i} N_{d,i} \left(\sum_{c,j} \widehat{M}_{(i,v,d),(j,u,c)} \right) / \sum_{d,i} N_{d,i} = N_{d,i} \left(\sum_{c,j} \widehat{M}_{(i,v,d),(j,u,c)} \right) / N_v$$

Analogously, we can aggregate the matrix $\widehat{M}_{(i,v,d),(j,u,c)}$ into $\widehat{M}_{d,c}$ over the age and SEP dimensions.

We then define the assortativity index of the matrix $\widehat{M}_{v,u}$ following Ref.[10] as follows:

$$\alpha = \widehat{M}_{v_1,v_1}/(\widehat{M}_{v_1,v_1} + \widehat{M}_{v_1,v_2}) + \widehat{M}_{v_2,v_2}/(\widehat{M}_{v_2,v_1} + \widehat{M}_{v_2,v_2})$$

i.e., as the sum of the proportion of contacts on the diagonal, with respect to the total number of contacts per row. If mixing is fully assortative, $\widehat{M}_{v_1,v_2} = \widehat{M}_{v_2,v_1} = 0$ and $\alpha = 2$; if mixing is completely disassortative, $\widehat{M}_{v_1,v_1} = \widehat{M}_{v_1,v_1} = 0$ and $\alpha = 1$; if mixing is homogeneous, i.e., proportional to the size of the groups, then $\widehat{M}_{v_1,v_1}/(\widehat{M}_{v_1,v_1} + \widehat{M}_{v_1,v_2}) = N_{v_1}/N$, and $\alpha = N_{v_1}/N + N_{v_2}/N = 1$.

In the main text, we showed the assortativity in the SEP and education level dimensions for each matrix in the set of matrices considered in **Fig. 3f**. In **Fig. S13**, we show the relative variation in the dominant eigenvalue of the matrix depending on the level of assortativity, computed with respect to a matrix assuming homogeneous mixing. We find that, for both SEP and education level, the dominant eigenvalue is on average slightly higher in the group of matrices with higher assortativity.

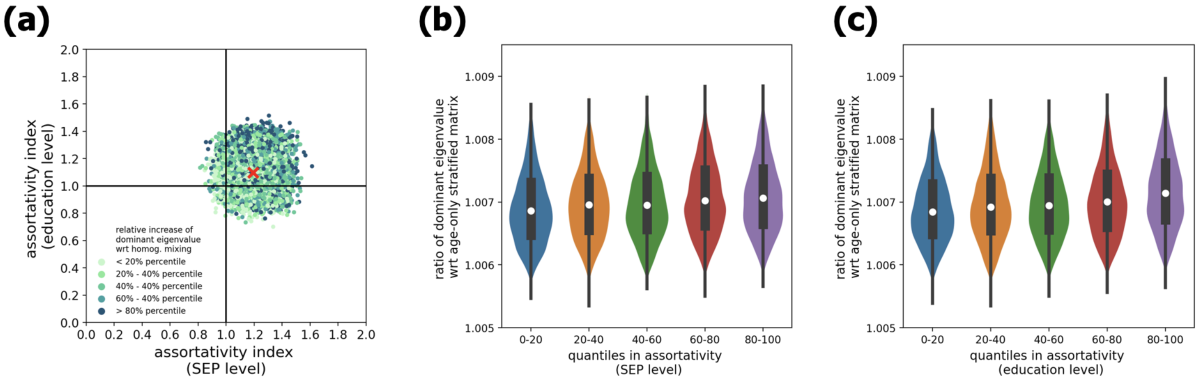

Figure S13. Matrix assortativity and dominant eigenvalue. (a) Scatter plot of assortativity index in two dimensions (education level and SEP level), color-coded based on ranges of relative increase in the dominant eigenvalue with respect to a homogeneous matrix. **(b-c)** Relative variation in the dominant eigenvalue (with respect to a matrix assuming homogeneous mixing in the SEP and education level dimension) as a function of assortativity in SEP (panel b) and education level (panel c).

3.4 Variation in dominant eigenvalue

Let $\widehat{M}_{hom}$ be the expanded contact matrix with homogeneous mixing in the SEP and education level dimension. In Ref.[11], it has been shown that (i) $\rho(\widehat{M}_{hom}) = \rho(M)$, where M is the age-stratified matrix, and (ii) $\rho(\widehat{M}) > \rho(\widehat{M}_{hom})$ for any matrix $\widehat{M}$ with non-homogeneous mixing on additional dimensions besides age (in our case, SEP and education level). Given the same transmission rate, modeling disease spread with an expanded contact $\widehat{M}$, i.e., accounting for heterogeneous mixing along

additional dimensions such as SEP and education level, would result in a higher reproductive number R_0 with respect to an age-stratified matrix M (or $\widehat{M}_{hom}$). In other words, the epidemic threshold (the critical transmission rate over which the epidemic takes off) would be higher for any heterogeneous $\widehat{M}$ compared to $\widehat{M}_{hom}$. On the other hand, given the same R_0, modeling the disease spread assuming the heterogeneous contact matrix $\widehat{M}$ would lead to a lower herd immunity threshold and a lower overall attack rate with respect to using M (or $\widehat{M}_{hom}$), as the additional heterogeneity in the contact patterns along SEP and education would further constrain the age-stratified epidemic[12].

In **Fig. 3f** in the main text, we showed the distribution of the ratio of the dominant eigenvalue of the expanded contact matrices with respect to an age-stratified matrix (neglecting the SEP and education level dimension). To quantify the relative role of the two social dimensions, here we show the variation in the dominant eigenvalue in a matrix stratified by age and SEP only, or by age and education level only. We found that introducing the education level dimension, while neglecting the SEP level, would contribute to a higher variation in the dominant eigenvalue with respect to an age-only stratified matrix, with a distribution of the relative increase centred between 0.23 % and 0.36 %. On the other hand, by introducing the stratification by SEP level, neglecting the education level, the distribution of the relative increase in the dominant eigenvalue is wider, ranging between 0.16% and 0.36%, with a median around 0.22%. The results are displayed in **Fig. S14**.

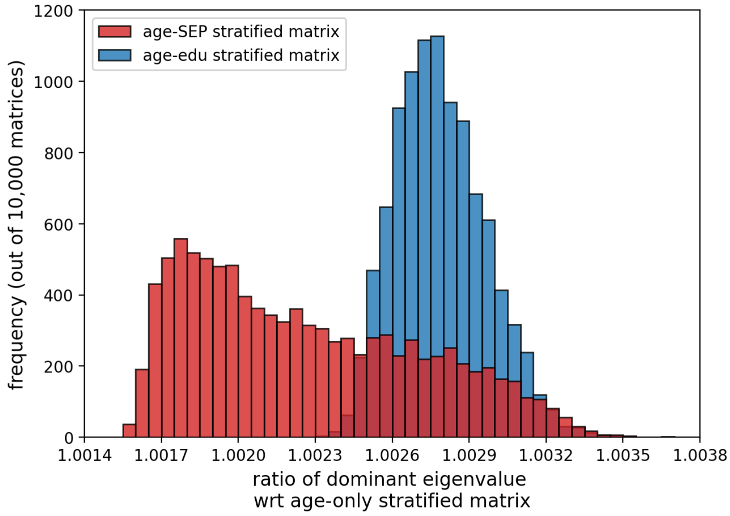

Figure S14. Impact of additional stratification compared to age-only stratified matrix. Distribution of the ratio of the dominant eigenvalue of expanded contact matrices that include age and one additional social dimension, with respect to an age-only stratified matrix (i.e., with homogeneous mixing in the SEP and education level dimensions). In red, results for matrices stratified by age and SEP level (neglecting stratification by education level). In blue, results for matrices stratified by age and education level (neglecting stratification by SEP level).

To compare the role of age and socio-economic dimensions, here we show the variation in the dominant eigenvalue in a matrix stratified by age only, by SEP only, by education level only, or by SEP and education level. The variation is computed with respect to a model with no structure (i.e., a constant contact rate for all groups, equal to the overall contact average). The results are displayed in **Fig. S15**. We found that incorporating the age dimension leads to a variation of 11%, much larger than the variation obtained by including SEP or education level or both socio-economic dimensions. This discrepancy is probably due to two concurrent factors: the fact that assortativity along age is much

stronger than assortativity along the socio-economic dimensions, and the fact that the stratification by age introduces more groups (4 age groups) with respect to stratification by SEP and education (two groups).

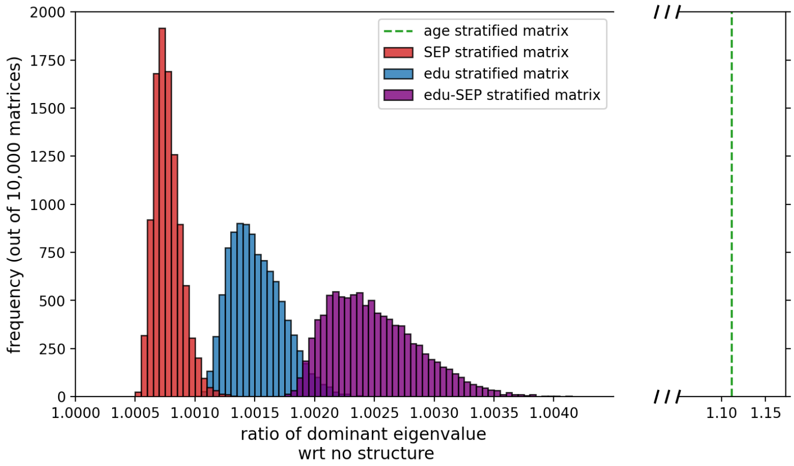

Figure S15. Impact of stratification in the contact matrix. Distribution of the ratio of the dominant eigenvalue of contact matrices, with respect to a model with no contact structure (i.e., a constant contact rate equal to the overall mean number of contacts). In red, results for matrices stratified by SEP level only (size 2x2); in blue, results for matrices stratified by education level only (size 2x2); in purple, results for matrices stratified by SEP and education level (size 4x4); the dashed vertical green line represent the value for the contact matrix stratified by age only (size 4x4).

4. Supplementary Results: epidemic control

4.1 Type-reproduction number

For heterogeneous mixing, the type-reproduction number T_g has been introduced as a measure of the control effort needed to contain the epidemic when targeting one specific group g of the population[13,14]. Practically, the type-reproduction number can be computed as the dominant eigenvalue of a matrix multiplication involving the next-generation matrix. Control can be implemented either through reduction in susceptibility (S-control, e.g. through vaccination) or through reduction in infectiousness (I-control, e.g. through reduction in contacts or shortening of the infectious period). Assuming an index case of type 1, the type-reproduction number T_1 denotes the cumulative number of infected hosts of type 1 resulting from all chains of infection, without another infected host of type 1 being allowed to reproduce. The type-reproduction number T_1 for one particular host group can be generalized to T_g to provide insights on the control effort needed to halt disease spread when targeting a subset of g host types. The epidemic is controlled if a proportion of group l greater than $1 - 1/T_g$ is permanently immune or fully isolated at the start of the epidemic. Thus, the quantity $1 - 1/T_g$ corresponds to the immunity threshold specific to some target group(s).

4.2 Epidemic scenario with homogenous susceptibility

In this section, we report the analysis of the effectiveness of targeted control strategies in an epidemic scenario with homogeneous susceptibility (**Fig. S16**), as done in the main text with the scenario with 50% reduction in susceptibility for children (**Fig. 5**).

In a scenario with homogeneous susceptibility, we found that children aged 0-14 play a major role in transmission (around ~50% contribution to R_0 accounting for both low SEP and high SEP), as shown in **Fig. 4** in the main text. Therefore, we would expect that a targeted strategy that includes children would be highly effective, while excluding children would hardly result in a successful control strategy. This is indeed what we found in **Fig. S16**. We found that a strategy targeting individuals with middle low education is effective for 100% of the contact matrices considered (**Fig. S16i**) and requires a low control effort (median 30%, 95% probability range 29% - 33%, **Fig. S11d**). Instead, a strategy targeted at individuals with a high education level (hence excluding all children) is never effective in preventing disease spread, regardless of the control effort (**Fig. S16f,i**). For a strategy targeted at individuals with high SEP and middle-low education level, we found a gradient in the control effort required as a function of the assortativity index in the education level and SEP dimensions (**Fig. S16h**), in line with what we found in the main text (**Fig. 5h**). The higher the assortativity in contacts in the additional social dimensions, the lower the chances that the strategy would be successful and the higher the control effort required to control the epidemic.

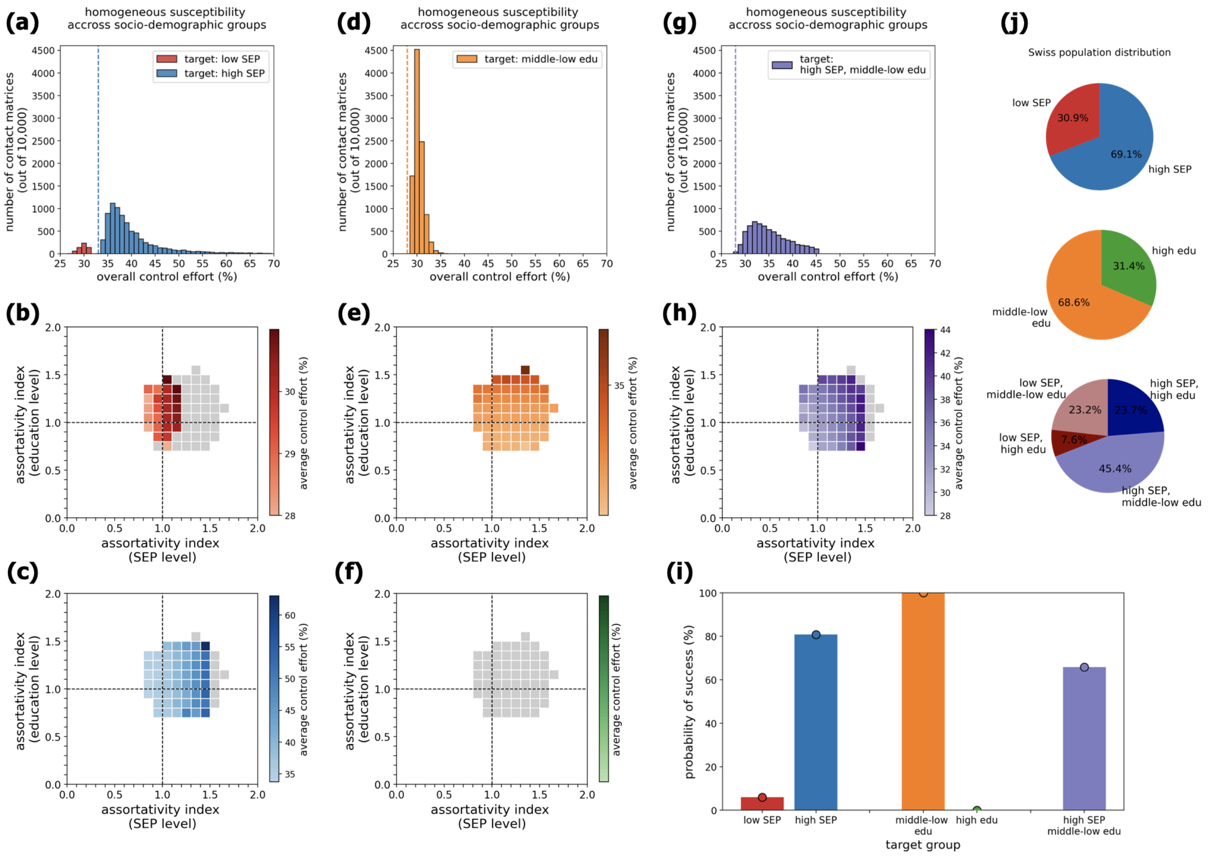

Figure S16. Effective targeted control strategies. Results for the epidemic scenario with homogeneous susceptibility across age and socio-economic groups. We considered a structured SIR epidemic model, stratified by age and socio-economic levels with heterogeneous mixing. We assumed $R_0 = 1.5$, and an average infectious period of 3 days. **(a)** Distribution of the overall control effort required by strategies targeted at individuals with low SEP (red) or with high SEP (blue), in the subset of matrices for which control was possible. Vertical dashed line represents control effort obtained from an epidemic model assuming an age-stratified matrix with homogeneous mixing in SEP and education level. **(b)** Assortativity levels in the SEP and education dimensions for the subset of matrices which allow effective control (colored cells) and those matrices for which the strategy would not be effective (grey cells). The color gradient indicates the average control effort required for a given range of assortativity. The strategy considered here is targeted at the low SEP group. **(c)** As in panel (b), but considering a strategy targeted at the group with high SEP. **(d)** Distribution of the overall control effort required by strategies targeted at individuals with middle-low (orange) or with high education level (green). **(e)** As in panel (b), but considering a strategy targeted at the group with middle-low education. **(f)** As in panel (b), but considering a strategy targeted at the group with high education. **(g)** Distribution of the overall control effort required by strategies targeted at individuals with high SEP and middle-low education level. **(h)** As in panel (b), but considering a strategy targeted at the group with high SEP and middle-low education level. **(i)** Probability of success of the targeted strategy, defined as the fraction of contact matrices for which there exists a critical control effort which allows epidemic control, out of a random sample of 10,000 synthetic expanded contact matrices with various assortativity levels. **(j)** Pie charts displaying the distribution of the population in three partitions, i.e., low SEP/high SEP (top), middle-low education/high education (center), and the combination of the two dimensions.

5. Supplementary Results: validation

We used the empirical data provided in Ref.[11] to validate our methodology. The authors in Ref.[11] provided an empirical contact matrix, stratified by 8 age groups and by 3 SES levels. Analogously to our main analysis, we aimed to consider 4 age groups and 2 SES levels. Hence, we aggregated the provided matrix in 4 age groups (0-14, 15-29, 30-69, 70+), and in 2 SES levels, low SEP (group 1) and high SES (merging group 2 and 3). We acknowledge that our methodology provides flexibility to be extended to a larger number of age groups and SES groups, but we leave this to future work.

Based on this stratification, we then computed the age-stratified matrix, the SES-stratified matrix, the fully stratified matrix, and the intermediate matrix where SES information is missing for contacts. Results are displayed in **Fig. S17**.

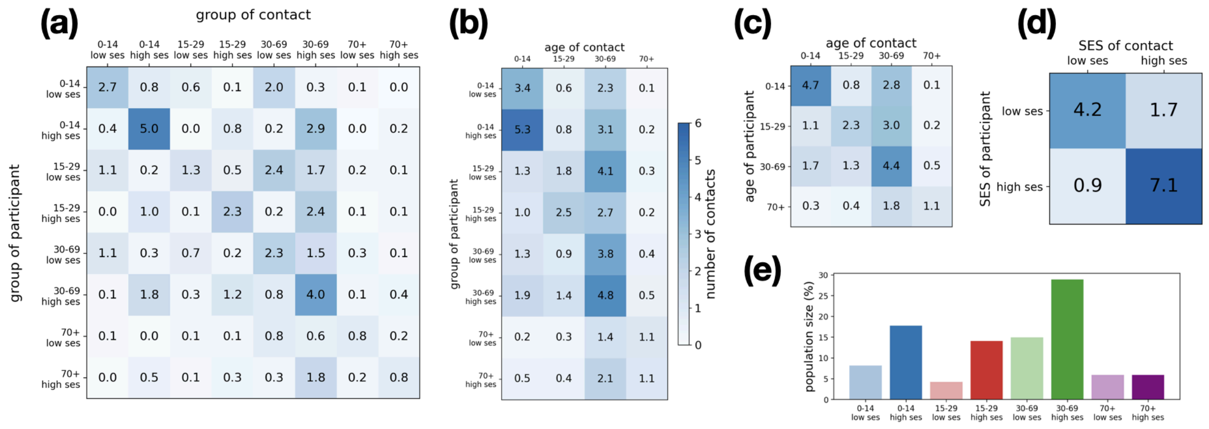

Figure S17. Empirical contact matrix stratified by age and SES. Data come from Ref[11]. We adapted the original matrix using a stratification with four age groups and two SES levels. **(a)** True expanded contact matrix fully stratified by age and SES. **(b)** Intermediate contact matrix. **(c)** Age-stratified contact matrix. **(d)** SES-stratified contact matrix. **(e)** Proportion of population in each group.

The matrix in **Fig. S17a** represents the "true" expanded matrix, which however is not available when SES information on contacts is missing. We now show that we can reconstruct the true expanded matrix from the intermediate contact matrix (**Fig. S17b**).

Given that we only have one additional socio-economic dimension (while in our original analysis we had both education level and SEP), the algorithm for the synthetic expansion requires a lower number of free parameters compared to the one presented in our main study.

For each of the four diagonal blocks (one for each age group), composed of 4 unknowns, we can write down 3 conditions, i.e. two conditions for the aggregation and one condition on the reciprocity. We then defined one assortativity parameter in the SES dimension, called $q^{i}_{v_1} \in (0, 1),\ v_1 = low\ SES$, representing the proportion of contacts that one individual with low SES level and age group i engages with the same group.

For each of the six coupled off-diagonal blocks with 8 variables, corresponding to a couple of age groups i and j with $i \neq j$, we can write 3 conditions on reciprocity, and 4 conditions on the aggregation. We then defined as free parameter $q^{i,j}_{v_1} \in (0, 1),\ v_1 = low\ SES$, similarly to what was done before, representing the proportion of contacts that one individual with low SES level and age group i engages with the group with low SES level and age j .

In total, we have 10 free parameters. We explored values ranging between 0 and 1 through random sampling from a uniform distribution, for each of the free parameters. We selected only those combinations leading to a positive contact matrix. The resulting selected distributions are illustrated in **Fig. S18**. For each parameter, we show the median value, the value expected from homogeneous/proportional mixing, and the values computed from the “true” empirical contact matrix. We verified that the latter values, when feeded into our algorithm, yield to the true empirical matrix, as expected.

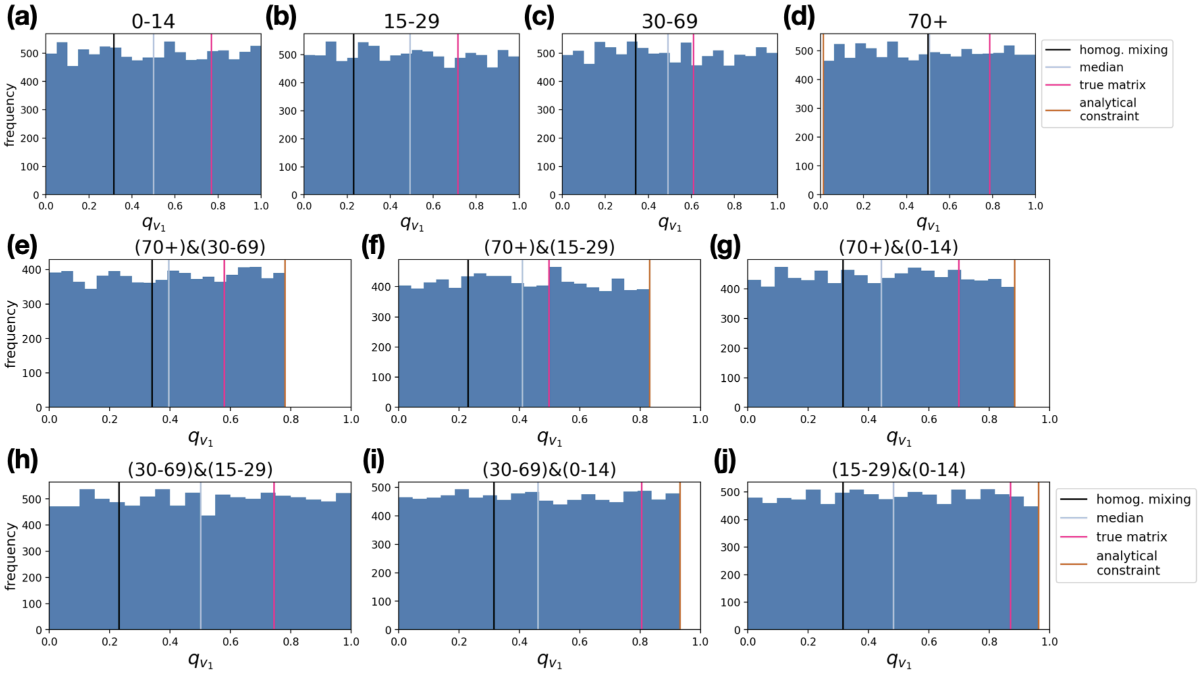

Figure S18. Parameter distributions. Frequency of values of the 10 free parameters of assortativity, which allow solving the system and derive a positive matrix. **(a-d)** Assortativity $q^{i}_{v_1}$ in the 4 diagonal blocks, one for each age group. **(e-j)** Assortativity $q^{i,j}_{v_1}$ in the 6 coupled off-diagonal blocks, for each age interaction. In each panel we show the median of the observed distribution (vertical line in light blue), the value expected from homogeneous mixing, i.e., mixing proportional to the size of the subgroups (vertical black line), and the value computed from the true expanded contact matrix (vertical pink line).

Finally, we generated 10,000 synthetic expanded contact matrices, by randomly sampling from the selected combinations of free parameters. We then computed the ratio in the dominant eigenvalue compared to an age-only stratified matrix, and the global assortativity index. We found that, as expected, our procedure allowed us to reconstruct ranges of assortativity values (**Fig. S19b**) compatible with the “true” matrix (which had SES assortativity equal to 1.6). From **Fig. S8** and **S19**, we can also observe that the assortativity of the “true” matrix is quite high within the range of compatible values.

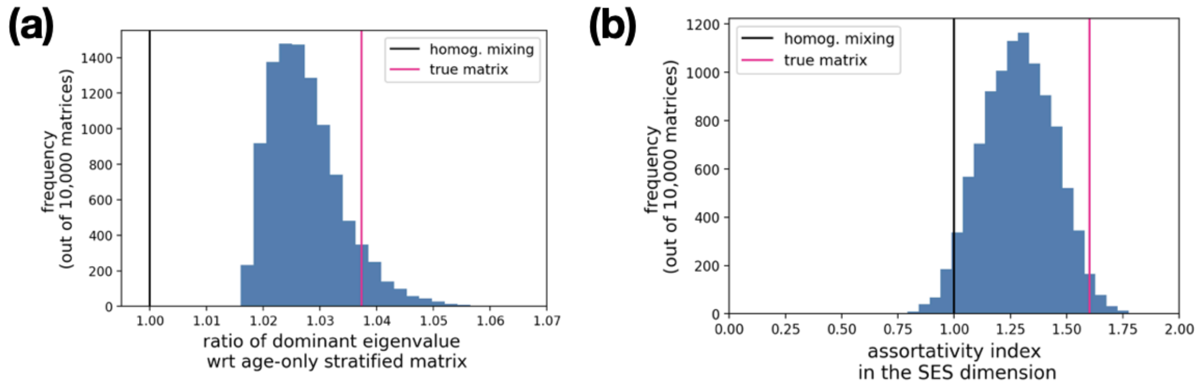

Figure S19. Validation of the reconstruction algorithm. (a) Distribution of the ratio of the dominant eigenvalue in the generated set of expanded contact matrices, compared to a matrix with homogeneous mixing in the SES dimension. **(b)** Distribution of the assortativity index in the SES dimension. In both panels, we show the results for 10,000 synthetic matrices. The values of assortativity index and dominant eigenvalue ratio of the true expanded contact matrix (**Fig. S17a**) are shown with a vertical pink line.

6. Supplementary Results: alternative definition of education level for children

In the main analysis, we classified all children and most young adults as middle-low education, to reflect their individual-based education level. However, one could argue that some of these children might be on their way to achieving high education, or live in otherwise high-education households, both of which might have an impact on their contact patterns.

To test this hypothesis, we carried out a sensitivity analysis where we assigned to survey participants younger than 18 the education level declared by their parents, who reported contacts on behalf of their children. In the multivariate regression analysis, we found that the rate ratio of contacts in children with high education compared to low education was not significant (**Fig S20**). This finding suggests that contacts in children are not highly influenced by the education level of their parents. All the other findings (e.g. association of contacts with SEP) are robust to this change in the definition of education level for children.

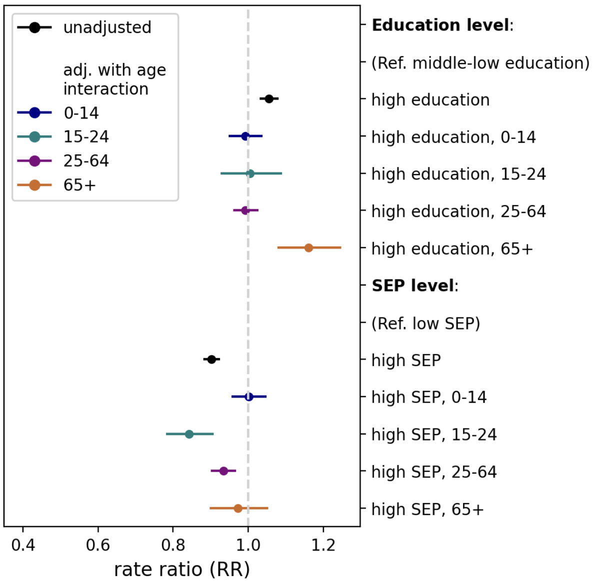

Figure S20. Results of the regression analysis. The rate ratio (RR) represents the relative change in the outcome, i.e., the average number of contacts of a participant in a given group compared to a group of reference. Black and colored dots represent the estimate obtained with a univariate and multivariate model respectively. Bars indicate 95% confidence intervals.

However, classifying some children as belonging to the high education group may impact the effectiveness of targeted control strategies, compared to the results of the main analysis. To assess this, we built a set of expanded contact matrices where children are distributed in groups with high or middle-low education. As in the main analysis, population data stratified by group are needed in order to adjust the matrix for reciprocity. To assign education level to young people based on the education level of their parents, we need to know which fraction of young individuals in Switzerland (further stratified by SEP) have parents with high or middle-low education. This information was not available in the official statistics. As an approximation, we assumed that this distribution is the same as the distribution of high / middle low education in the general adult population. However, we note that this distribution may differ from the one observed in the general population of adults, which may or may not have children, and the number of children may also depend on the education level.

We distributed the number of low-SEP children (0-14 y.o.) and low-SEP young adults (15-24 y.o., with middle-low education) into middle-low and high education, based on the fraction of low SEP adults with middle-low or high education. Similarly, we distributed the number of high-SEP children and high-SEP young adults (with middle-low education) into middle-low and high education, based on the fraction of high-SEP adults with middle-low or high education. As a result, the population sizes in each group were updated as shown in **Fig. S21** (to be compared with **Fig. 1a** for the main analysis).

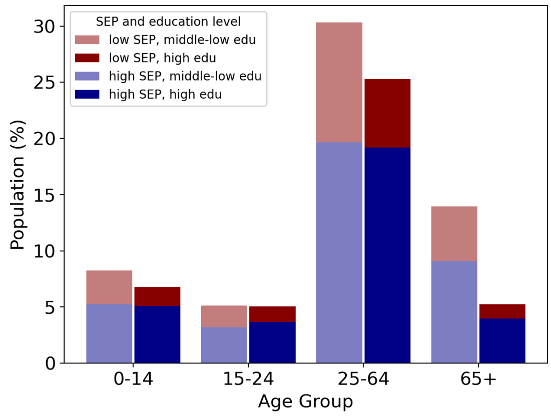

Figure S21. Swiss population. Population size (in %) in Switzerland by age group (x-axis), SEP level (red/blue color), and education level (lighter/darker shade). Education level for individuals younger than 18 (with a given SEP level) was assigned based on the observed fraction of high or middle-low education in the group 25-64 y.o. with the corresponding SEP level.

We then followed analogous steps as in the main analysis. First, we estimated the intermediate contact matrix (**Fig. 22a**), and generated a set of synthetic expanded contact matrices compatible with the observed matrix. We illustrate one of the expanded synthetic matrices in **Fig. 22b**. We computed the assortativity index and the ratio of the dominant eigenvalue compared to an age-only stratified matrix (**Fig. 22c,d**). We found that the assortativity plane compatible with the data had a similar shape compared to the main analysis, with a slightly bigger area spanning in the bottom right quadrant (lower values in assortativity index). This makes sense as distributing children in two groups - rather than forcing them to be in the same education level - allows to derive compatible matrices with less assortative contacts. The variation observed in R0 was slightly larger than the main analysis, but still quite small (around 2% rather than 0.7%).

Second, we compute the relative attack rate and the contribution to R0 for each group. Results are shown in **Fig. S23** (to be compared with **Fig. 4**). We found that the relative attack rates did not substantially change compared to the main analysis. In terms of contributions to R0, children and young adults in middle-low education contribute less in the sensitivity analysis, compared to the main analysis, as expected as their population size is reduced. For the other age groups, results were robust compared to the main analysis.

Finally, we quantified the effectiveness of targeted control strategies, in the scenario with reduced susceptibility in children. Results are shown in **Fig. S24** (to be compared with **Fig. 5**). As expected, the higher the assortativity in the contacts, the higher the control effort of a targeted strategy required to control the epidemic. A notable difference in the sensitivity analysis compared to the main analysis is the effectiveness of targeting individuals in the group of high SEP and high education. Indeed, we found a set of contact matrices (however very few, less than 5%), for which a control effort around 30% would be effective. In contrast, in the main analysis we found that control was never achievable regardless of the assortativity in contacts. However, this is expected given that the size of the target group (high SEP and high education) is larger in the sensitivity analysis (32%) because it includes also a fraction of the children group, compared to the main analysis (24%) where children were

excluded as they were all classified as middle-low education. In general, with this definition of education level for children, we found that strategies targeted at individuals with high education were more effective compared to the main analysis.

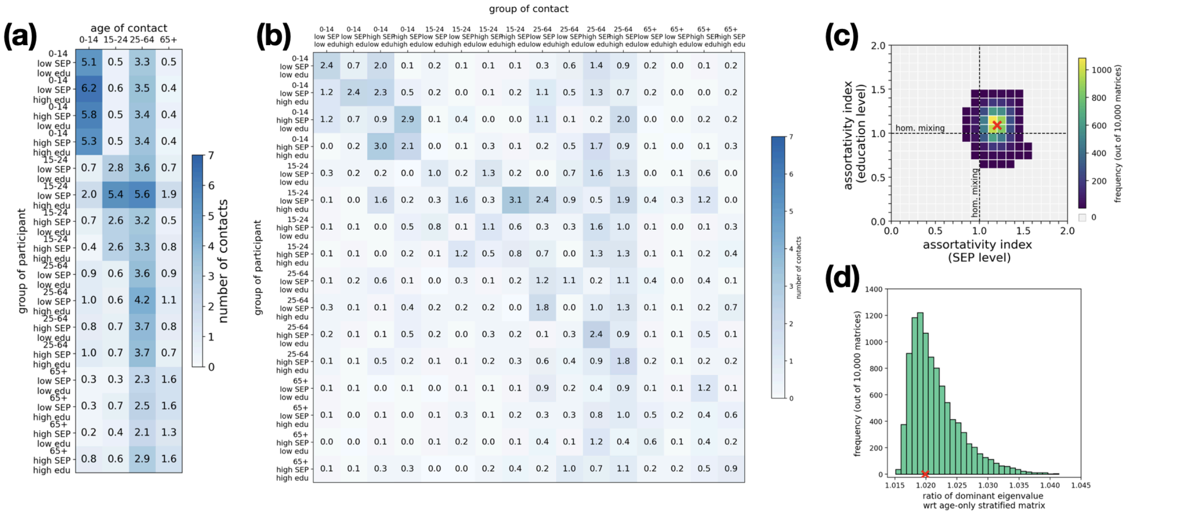

Figure S22. Expanded contact matrices. (a) Intermediate contact matrix stratified by age, SEP and education level on the participants' side, adjusted for reciprocity, informed from the available data. **(b)** One example of a fully expanded synthetic contact matrix, compatible with the intermediate matrix under aggregation. **(c)** Heatmap of the frequency of values of the assortativity index in the two dimensions (education level on y axis and SEP level on x axis). Grey cells indicate ranges of assortativity not observed in our set of expanded contact matrices. **(d)** Distribution of the ratio of the dominant eigenvalue of the expanded contact matrices compared to a matrix with homogeneous mixing in the SEP and education level dimensions. In both panels (c) and (d), we show the results for 10,000 synthetic matrices.

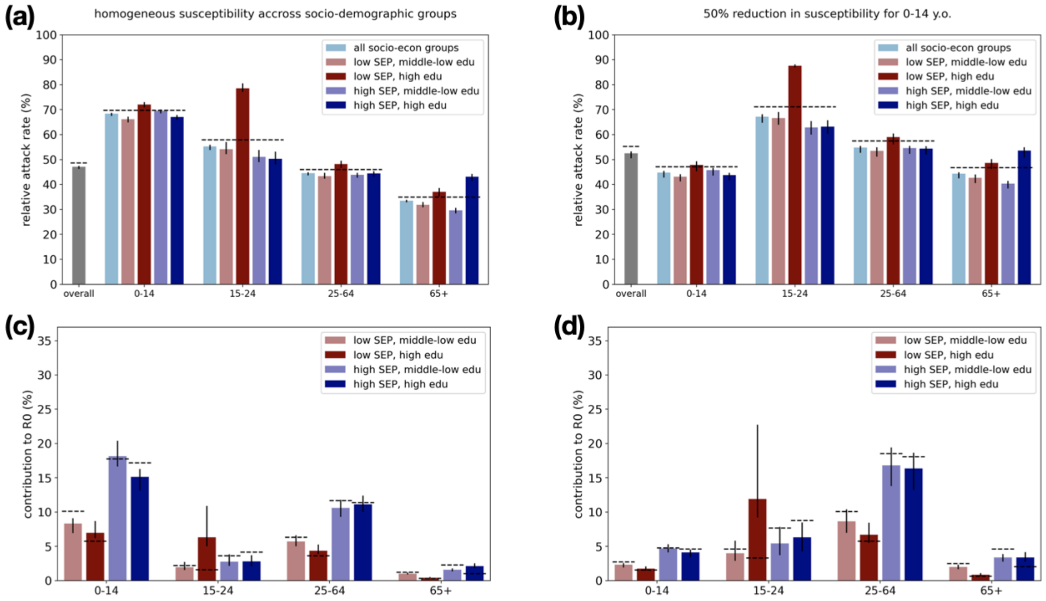

Figure S23. Epidemic spread in absence of control strategies. (a) Relative attack rate for each group, i.e., fraction of the cumulative number of infected individuals over the size of the group. **(b)** Proportional contribution to R0 of each group. In both panels, horizontal dashed lines indicate results obtained from a reference epidemic model assuming an age-stratified matrix with homogeneous mixing in SEP and education level. **(c-d)** As in (a-b), but assuming a lower susceptibility for the youngest age group of children (0-14 y.o) with a 50% reduction compared to the other groups.

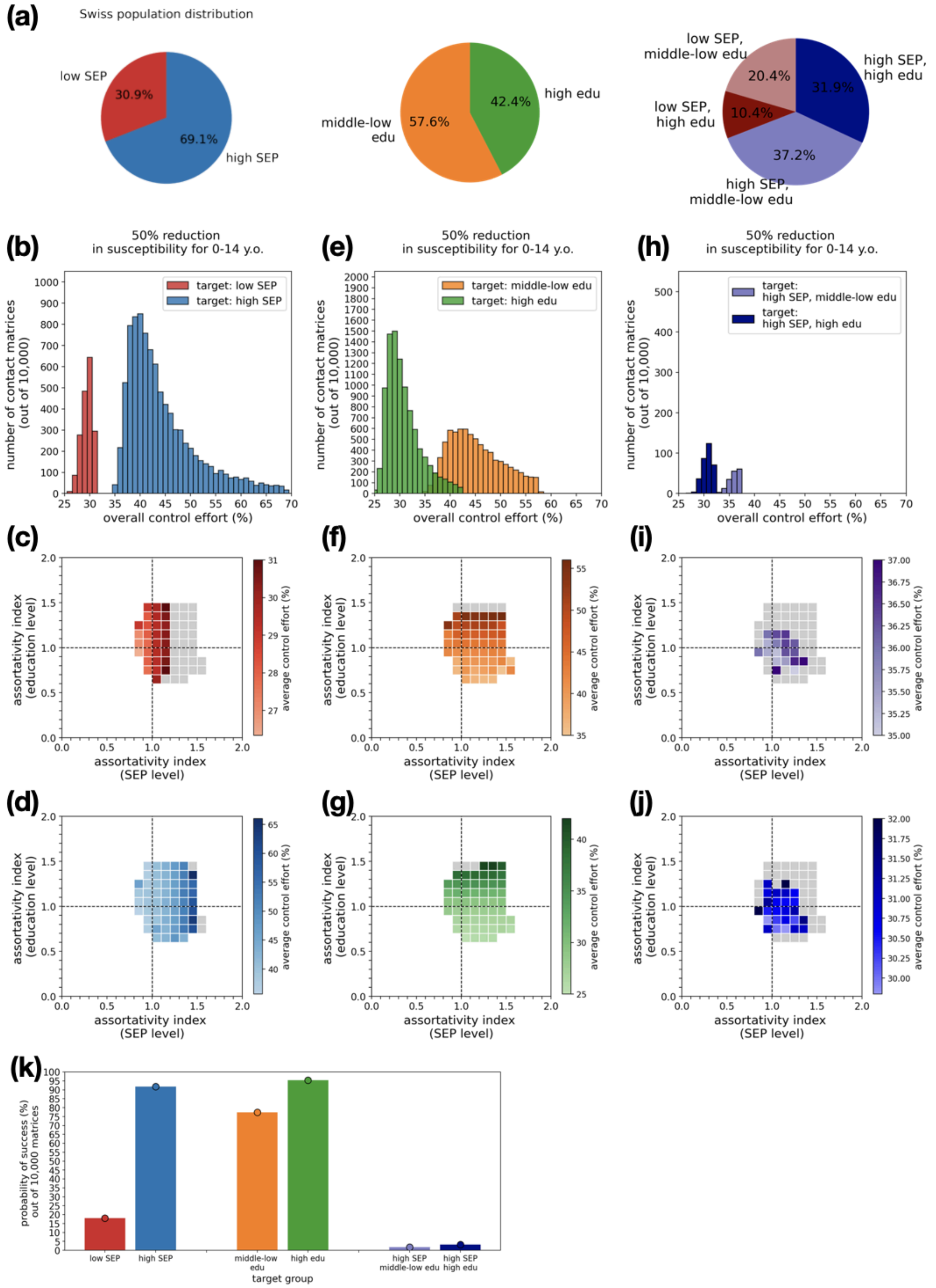

Figure S24. Effective targeted control strategies. Results for the epidemic scenario with reduced susceptibility for children. **(a)** Pie charts displaying the distribution of the population in three partitions, i.e., low SEP/high SEP (left), middle-low education/high education (center), and the combination of the two dimensions (right). **(b)** Distribution of the overall control effort required by strategies targeted at individuals with low SEP (red) or with high SEP (blue), in the subset of matrices for which control was possible. **(c)** Assortativity levels in the SEP and education dimensions for the subset of matrices which allow effective control (colored cells) and those matrices for which the strategy would not be effective (grey cells). The color gradient indicates the average control effort

required for a given range of assortativity. The strategy considered here is targeted at the low SEP group. **(d)** As in panel (c), but considering a strategy targeted at the group with high SEP. **(e)** Distribution of the overall control effort required by strategies targeted at individuals with middle-low (orange) or with high education level (green). **(f)** As in panel (c), but considering a strategy targeted at the group with middle-low education. **(g)** As in panel (c), but considering a strategy targeted at the group with high education. **(h)** Distribution of the overall control effort required by strategies targeted at individuals with high SEP and middle-low education level, or high SEP and high education. **(i)** As in panel (c), but considering a strategy targeted at the group with high SEP and middle-low education level. **(j)** As in panel (c), but considering a strategy targeted at the group with high SEP and high education level. **(k)** Probability of success of the targeted strategy, defined as the fraction of contact matrices for which there exists a critical control effort allowing epidemic control, out of a random sample of 10,000 synthetic expanded contact matrices with various assortativity levels.

Supplementary References

1. Reichmuth, M. L. *et al.* Social contacts in Switzerland during the COVID-19 pandemic: insights from the CoMix study. *Epidemics* 100771 (2024) doi:10.1016/j.epidem.2024.100771.
2. State Secretariat for Education, Research and Innovation (SERI). Swiss Education System. https://www.sbfi.admin.ch/sbfi/en/home/bildung/bildungsraum-schweiz/das-duale-system.html.
3. Hale, T. *et al.* A global panel database of pandemic policies (Oxford COVID-19 Government Response Tracker). *Nat. Hum. Behav.* **5**, 529–538 (2021).
4. Google. COVID-19 Community Mobility Report. *COVID-19 Community Mobility Report* https://www.google.com/covid19/mobility?hl=en.
5. Federal Statistical Office. Population and household statistics (STATPOP), geodata 2022. https://www.bfs.admin.ch/asset/en/27965868.
6. Federal Statistical Office. ThemaKart map boundaries - Set 2023. https://www.bfs.admin.ch/asset/en/24025646.
7. Panczak, R., Berlin, C., Voorpostel, M., Zwahlen, M. & Egger, M. The Swiss neighbourhood index of socioeconomic position: update and re-validation. *Swiss Med. Wkly.* **153**, 40028–40028 (2023).
8. Federal Office of Topography (swisstopo). Population (residents). https://map.geo.admin.ch/#/map?lang=en&center=2533675.22,1170896.93&z=2.479&topic=ech&layers=ch.swisstopo.zeitreihen@year=1864,f;ch.bfs.gebaeude_wohnungs_register,f;ch.bav.haltestellen-oev,f;ch.swisstopo.swisstlm3d-wanderwege,f;ch.bfs.volkszaehlung-bevoelkerungsstatistik_einwohner@year=2021;ch.bak.schutzgebiete-unesco_weltkulturerbe,f&bgLayer=ch.swisstopo.pixelkarte-farbe.

9. Manna, A., Koltai, J. & Karsai, M. Importance of social inequalities to contact patterns, vaccine uptake, and epidemic dynamics. *Nat. Commun.* **15**, 4137 (2024).

10. Garnett & Anderson. Factors controlling the spread of HIV in heterosexual communities in developing countries: patterns of mixing between different age and sexual activity classes. *Philos. Trans. R. Soc. Lond. B. Biol. Sci.* **342**, 137–159 (1993).

11. Manna, A., Dall'Amico, L., Tizzoni, M., Karsai, M. & Perra, N. Generalized contact matrices for epidemic modeling. *Sci. Adv.* **10**, (2024).

12. Britton, T., Ball, F. & Trapman, P. A mathematical model reveals the influence of population heterogeneity on herd immunity to SARS-CoV-2. *Science* **369**, 846–849 (2020).

13. Roberts, M. G. & Heesterbeek, J. A. P. A new method for estimating the effort required to control an infectious disease. *Proc. R. Soc. Lond. B Biol. Sci.* **270**, 1359–1364 (2003).

14. Heesterbeek, J. A. P. & Roberts, M. G. The type-reproduction number T in models for infectious disease control. *Math. Biosci.* **206**, 3–10 (2007).
