# Supplementary material for: Individual and neighborhood based socioeconomic factors relevant for contact behaviour and epidemic control
Source: Commun Med (Lond). 2025 Dec 11;6:26. doi: 10.1038/s43856-025-01282-y (PMC12808736; doi:10.1038/s43856-025-01282-y)
Supplement: Supplementary file 3 — Description of Additional Supplementary files [file 43856_2025_1282_MOESM3_ESM.docx]

**Description of Additional Supplementary Files**

File name: Supplementary Data 1-5

Description: Source data for the figures
